# The osteoprotective role of USP26 in coordinating bone formation and resorption

**DOI:** 10.1038/s41418-021-00904-x

**Published:** 2022-01-29

**Authors:** Changwei Li, Minglong Qiu, Leilei Chang, Jin Qi, Lianfang Zhang, Bernhard Ryffel, Lianfu Deng

**Affiliations:** 1 Department of Orthopedics, Shanghai Key Laboratory for Prevention and Treatment of Bone and Joint Diseases, Shanghai Institute of Traumatology and Orthopedics, Ruijin Hospital, Shanghai Jiaotong University School of Medicine, 197 Ruijin 2nd Road, Shanghai, 200025 China; 2 Department of Orthopedics, First Affiliated Hospital of Soochow University, Suzhou, Jiangsu China; 3 Laboratory of Experimental and Molecular Immunology and Neurogenetics (INEM), UMR 7355 CNRS, University of Orleans, ArtImmune SAS, F-45071 Orleans, France

**Keywords:** Deubiquitylating enzymes, Deubiquitylating enzymes, Endocrine system and metabolic diseases, Immunopathogenesis

## Abstract

Bone homeostasis is maintained through a balance of bone formation by osteoblasts and bone resorption by osteoclasts. Ubiquitin-specific proteases (USPs) are involved in regulating bone metabolism by preserving bone formation or antagonizing bone resorption. However, the specific USPs that maintain bone homeostasis by orchestrating bone formation and bone resorption simultaneously are poorly understood. Here, we identified USP26 as a previously unknown regulator of bone homeostasis that coordinates bone formation and resorption. Mechanistically, USP26 stabilizes β-catenin to promote the osteogenic activity of mesenchymal cells (MSCs) and impairs the osteoclastic differentiation of bone myelomonocytes (BMMs) by stabilizing inhibitors of NF-κBα (IκBα). Gain-of-function experiments revealed that *Usp26* supplementation significantly increased bone regeneration in bone defects in aged mice and decreased bone loss resulting from ovariectomy. Taken together, these data show the osteoprotective effect of USP26 via the coordination of bone formation and resorption, suggesting that USP26 represents a potential therapeutic target for osteoporosis.

## Introduction

Bone homeostasis is maintained through a balance of bone formation by osteoblasts and bone resorption by osteoclasts [1]. Osteoblasts, which originate from mesenchymal precursors, are responsible for the deposition of new bone matrix and its mineralization, whereas osteoclasts are giant multinucleated cells that originate from the myelomonocytic lineage and are uniquely capable of resorbing the mineralized matrix [2]. An imbalance between the activity of osteoblasts and osteoclasts leads to improper bone formation and resorption, which underlies the pathogenesis of osteoporosis, the most common skeletal disease. Therefore, a better understanding of the balancing mechanisms is crucial for the development of therapeutic agents [3].

Bone formation is linked to resorption through coupling factors, and this coupling limits the effectiveness of current therapies for the treatment of osteoporosis. Antiresorptives targeting osteoclasts can induce a decrease in osteoblast activity, and the ability of the parathyroid hormone agonist teriparatide to promote bone formation is partially counterbalanced by increased osteoclast resorptive activity [4, 5]. Thus, identification of molecules that simultaneously enhance bone formation and suppress bone resorption is eagerly awaited.

Ubiquitin-dependent proteolysis is crucial for the fine-tuning of osteoblast and osteoclast lineage differentiation [6, 7]. The ubiquitination system is an enzymatic cascade that adds ubiquitin chains to target proteins, thereby directing their degradation. Polyubiquitin chains attached to target proteins can be edited or removed by deubiquitinating enzymes [8]. The ubiquitin-specific protease (USP) family, which consists of more than 50 known members, is the largest of five families of deubiquitinases [1]. Although several USPs, such as USP1 and USP34, are involved in regulating bone formation by facilitating mesenchymal cell (MSC) osteogenesis, or antagonizing osteoclast differentiation of bone myelomonocytes (BMMs) [1, 9], very little is known about which USPs maintain bone homeostasis by orchestrating bone formation and bone resorption simultaneously.

Here, we screened members of the USP family and identified USP26 as a previously unknown regulator of bone homeostasis via the coordination of bone formation and resorption. Mechanistically, USP26 stabilizes β-catenin to promote the osteogenic activity of MSCs and impairs osteoclastic differentiation of BMMs by stabilizing inhibitors of NF-κBα (IκBα). Gain-of-function experiments revealed that USP26 significantly increased bone regeneration in aged mice and decreased bone loss resulting from ovariectomy. Our data show the osteoprotective effect of USP26 via the coordination of bone formation and resorption and suggest that USP26 represents a novel therapeutic target for osteoporosis.

## Results

### *Usp26* facilitates osteoblastic differentiation of MSCs and impairs osteoclastic differentiation of BMMs

**Fig. 1:**
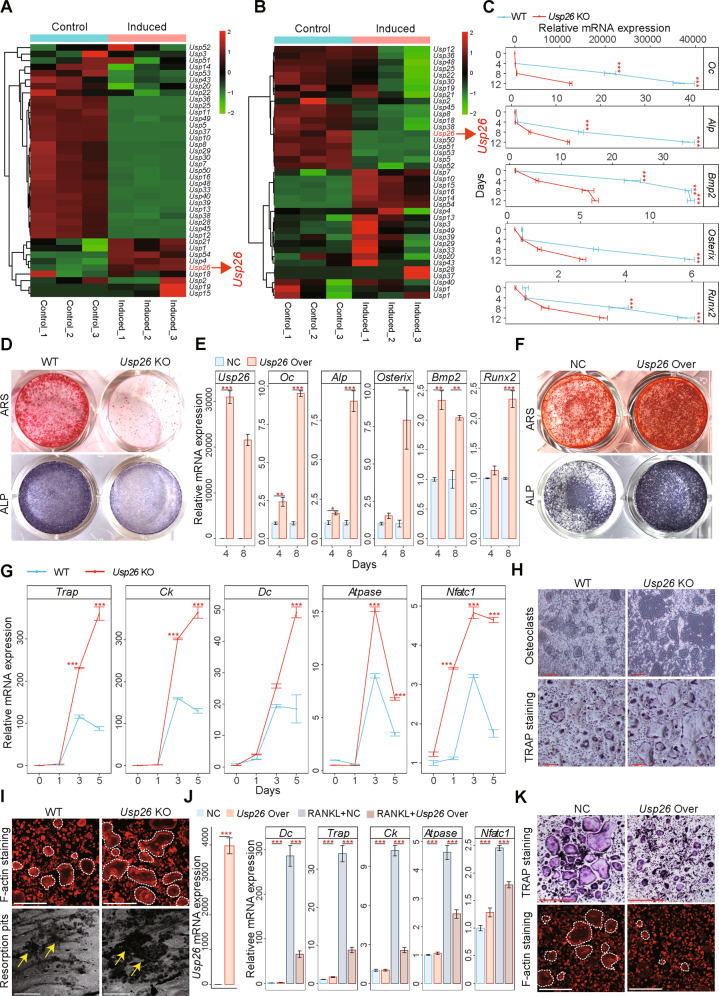
*Usp26* regulates osteogenic differentiation of MSCs and osteoclastic differentiation of BMMs. **A** Heatmap of USPs mRNA expression in MSCs after 8 days of osteoblastic differentiation. **B** Heatmap of USPs mRNA expression in BMMs after 3 days of osteoclastic differentiation. **C** Time curve of *Oc*, Alp, *Bmp2*, *Osterix*, and *Runx2* expression in MSCs obtained from *Usp26*^−/−^ and littermate controls after osteoblastic differentiation. **D** Representative images of ARS and ALP staining of MSCs obtained from *Usp26*^−/−^ and littermate controls after 8 days of osteoblastic differentiation. **E** Quantification analysis of *Usp26*, *Oc*, *Alp*, *Osterix*, *Bmp2*, and *Runx2* expression in MSCs after 4 and 8 days of osteoblastic differentiation with or without *Usp26* overexpression. **F** Representative images of ARS and ALP staining in MSCs after 8 days of osteoblastic differentiation with or without *Usp26* overexpression. **G** Time curve of *Trap*, *Ck* (*Cathepsin k*), *Dc* (*DC-STAMP*), *Atpase* (*V-atpase α3*), and *Nfatc1* expression in BMMs obtained from *Usp26*^−/−^ and WT littermate mice after osteoclastic differentiation. Representative images of multinuclear osteoclasts formation and its TRAP staining (**H**), F-actin staining and bone resorption assay (**I**) of BMMs obtained from *Usp26*^−/−^ and WT littermate mice after 5 days of osteoclastic differentiation. Dotted yellow lines indicate the resorption pits. **J** Quantification analysis of *Usp26*, *Dc*, *Trap*, *Ck*, *Atpase*, and *Nfatc1* expression in BMMs after 3 days of osteoclastic differentiation with or without *Usp26* overexpression. **K** Representative images of TRAP and F-actin staining in BMMs after 5 days of osteoclastic differentiation with or without *Usp26* overexpression. Scale bars represent 200 μm. *Usp26* Over and NC represent cells transfected with *Usp26* overexpression lentivirus and its negative controls. *P < 0.05, **P < 0.01, ***P < 0.001. *P* values were analyzed by two-tailed *t* tests in **E**, one-way ANOVA in **J**, and two-way ANOVA in **C** and **G**. All data are representative of two to three independent experiments.

To investigate the potential roles of USPs in coordinating bone formation and resorption, we first profiled the expression of 54 known USPs in mouse MSCs and BMMs after osteoblastic and osteoclastic differentiation, respectively. Quantitative reverse transcriptase-polymerase chain reaction (RT-qPCR) revealed that in addition to the undetected USPs, *Usp26* together with *Usp4*, *Usp18* and *Usp21* were specifically upregulated after osteoblastic differentiation (Fig. 1A and Fig. S1A), but were downregulated after osteoclastic differentiation (Fig. 1B and Fig. S1B). USP4 has been proven to antagonize osteoblastogenesis [10]. We next sought to examine the roles of USP26, USP18 and USP21 in the osteoblastic differentiation of MSCs. MSCs isolated from *Usp26*^−/−^ mice and their wild-type (WT) littermate controls were cultured in osteogenic media for 0, 4, 8, and 12 days. RT-qPCR demonstrated that osteogenic induction upregulated osteogenesis-related genes, including *osteocalcin* (*Oc*)*, alkaline phosphatase* (*Alp*), *bone morphogenetic protein-2* (*Bmp2*), *osterix*, and *runt-related transcription factor 2* (*Runx2*), in a time-dependent manner in MSCs from WT mice. However, the expression of these genes was substantially reduced in the absence of *Usp26* (Fig. 1C). In addition, ALP staining and alizarin red S (ARS) staining results showed that ALP activity and extracellular matrix mineralization were also markedly reduced in *Usp26*^−/−^ MSCs (Fig. 1D). Gain-of-function experiments showed that *Usp26* overexpression significantly increased osteoblastic gene expression, ALP activity and extracellular matrix mineralization (Fig. 1E, F). To detect whether USP18 and USP21 are required for osteogenic differentiation, *Usp18* and *Usp21* were knocked down in MSCs with short hairpin RNAs (shRNAs). The results showed that *Usp18* knockdown significantly increased the osteogenic potential of MSCs, whereas *Usp21* deletion decreased the osteoblastic differentiation of MSCs (Fig. S1C–F).

USP18 has been shown to impair the osteoclastic activities of BMMs [11]. We next sought to detect the role of USP26, USP4, and USP21 in osteoclastic differentiation. BMMs isolated from *Usp26*^−/−^ mice and their littermate controls were cultured in osteoclastic medium. *Usp26* deficiency resulted in a significant upregulation of osteoclastic genes, including *dendritic cell-specific transmembrane protein* (*DC-STAMP*), *tartrate-resistant acid phosphatase* (*Trap*), *cathepsin k* (*Ck*), *V-atpase α3* (*Atpase*), and *nuclear factor of activated T cell cytoplasmic 1* (*Nfatc1*) (Fig. 1G). The enhanced effect of *Usp26* deficiency on osteoclastic differentiation was further demonstrated by enhanced TRAP staining with an increased number of multinucleate osteoclasts (Fig. 1H). Furthermore, F-actin ring formation and bone resorption pit assays demonstrated that *Usp26* deficiency significantly enhanced mature osteoclast formation (Fig. 1I). Conversely, *Usp26* overexpression significantly dampened the expression of osteoclastic genes and inhibited the formation of multinucleate osteoclasts (Fig. 1J, K). In contrast to *Usp26*, *Usp21* knockdown in BMMs significantly decreased osteoclastic gene expression and dampened the formation of multinucleate osteoclasts (Fig. S1G, H), whereas *Usp4* knockdown had no significant influence on the osteoclastic activity of BMMs (Fig. S1I, J). Collectively, these results indicated that USP26 is a unique USP that facilitates osteoblastic differentiation of MSCs and impairs osteoclastic differentiation of BMMs, motivating further investigations on the potential role of USP26 in bone formation and resorption.

### *Usp26*^−/−^ mice show decreased bone formation and increased bone resorption

**Fig. 2:**
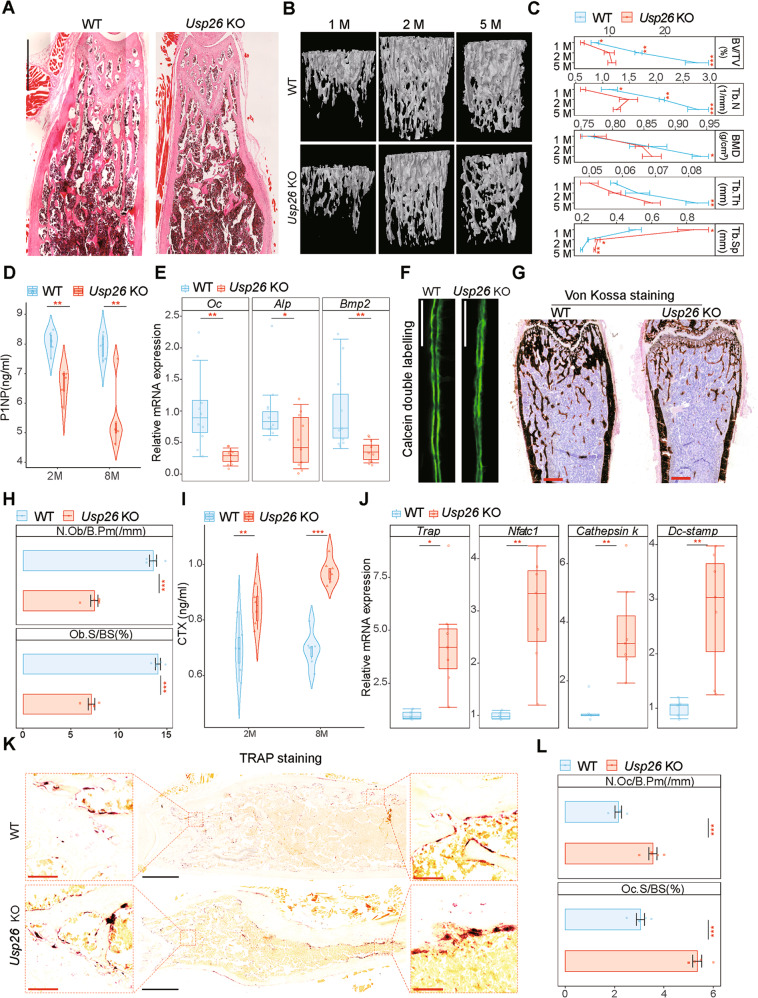
*Usp26*^−/−^ mice have decreased bone formation and increased bone resorption. **A** Representative images of H&E staining of femurs of 5-month-old *Usp26*^−/−^ mice and the littermate controls (*n* = 5). Scale bar represents 1000 μm. **B** Representative micro-CT images of trabecular bone from the femoral metaphysis of 1-, 2- and 5-month-old *Usp26*^−/−^ mice and their WT littermates (*n* = 5). **C** Cancellous bone volume (BV/TV, %), trabecular number (Tb.N, 1/mm), trabecular separation (Tb.Sp, mm), trabecular thickness (Tb.Th, mm), and trabecular bone mineral density (BMD, g/cm^3^) were determined by micro-CT analysis (*n* = 5). **D** Quantification analysis PINP in serum of 2- and 8-month-old *Usp26*^−/−^ mice and their WT littermates with ELISA (*n* = 5). **E** Quantification analysis of *Oc*, *Alp*, and *Bmp2* expression in femurs of 5-month-old *Usp26*^−/−^ mice and their WT littermates (*n* = 10). **F** New bone formation was determined by calcein double labeling (*n* = 5). Scale bars represent 200 μm. **G**, **H** Von Kossa staining and histomorphometrical analysis of femurs of 5-month-old *Usp26*^−/−^ mice and their WT littermates (*n* = 5). Scale bars represent 500 μm. **I** Quantification analysis CTX-I in serum of 2- and 8-month-old *Usp26*^−/−^ mice and their WT littermates with ELISA (*n* = 6). **J** Quantification analysis of *Trap*, *Ck*, *Nfatc1*, and *Dc* expression in femurs of 5-month-old *Usp26*^−/−^ mice and their wild-type littermates (*n* = 5 or 7). **K**, **L** TRAP staining and histomorphometrical analyses of femurs of 5-month-old *Usp26*^−/−^ mice and their WT littermates (*n* = 5). Red and black bars represent 25 and 100 μm, respectively. *P < 0.05, **P < 0.01, ***P < 0.001. *P* values were analyzed by two-way ANOVA in **C**, two-tailed *t* tests in **D**, **I**, **E**, **H**, **J**, and **L**. All data are representative of two to three independent experiments.

First, bone mass was compared between *Usp26*^−/−^ mice and their littermate controls. Hematoxylin–eosin (H&E) staining revealed that trabecular bone was significantly less in the femurs of 5-month-old *Usp26*^−/−^ mice than in those of littermate controls (Fig. 2A). Further analysis of trabecular bone from the distal femur metaphysis by micro-quantitative computed tomography (micro-CT) demonstrated that *Usp26*^−/−^ mice had decreased cancellous bone volume/tissue volume (BV/TV), a lower trabecular number (Tb. N) and increased trabecular separation (Tb. Sp) from 1 to 5 months of age (Fig. 2B, C and Fig. S2A). The trabecular thickness (Tb. Th) and bone mineral density (BMD) of *Usp26*^−/−^ mice were significantly lower than those of littermate controls at 5 months of age (Fig. 2C). In addition, *Usp26* deficiency also resulted in decreased cortical BV/TV, cortical BMD, cortical thickness (Ct. Th), and total cross-sectional cortical bone area (B. Ar) and increased cortical porosity (Ct. Po) (Fig. S2B, C). Biomechanical properties determined via three-point bending revealed that less force was required for femoral fracture of *Usp26*^−/−^ mice (Fig. S2D) and that the elastic modulus was also decreased because of the increased cortical porosity (Fig. S2D). Taken together, these data demonstrate that *Usp26* deficiency leads to low bone mass and poor bone strength.

To detect whether the low bone mass in *Usp26*^−/−^ mice resulted from decreased bone formation, the serum level of the bone formation marker N-terminal propeptide of type I procollagen (PINP) was first assessed via enzyme-linked immunosorbent assay (ELISA). The results showed that PINP levels were reduced in both 2- and 8-month-old *Usp26*^−/−^ mice compared with littermate controls (Fig. 2D). Moreover, the expression of osteogenic markers, including *Oc*, *Alp*, *Bmp2*, and *osterix*, was significantly decreased in the femoral bone samples of 5-month-old *Usp26*^−/−^ mice, as detected by RT-qPCR and immunohistochemical staining analyses (Fig. 2E and Fig. S3A). In addition, double calcein labeling and Von Kossa staining showed a decreased bone formation rate and decreased mineralization level in *Usp26*^−/−^ mice compared with littermate controls (Fig. 2F, G). The histomorphometric analysis revealed a consistent, significant reduction in osteoblast numbers (N.Ob/B. Pm (/mm)) and osteoblast surfaces (Ob. S/BS (%)) (Fig. 2H). These data demonstrate decreased bone formation in *Usp26*^−/−^ mice.

Bone homeostasis is dependent on the coupling of bone formation and resorption [12]. The ELISA results showed that *Usp26* deficiency significantly increased the concentration of C-telopeptide of type I collagen (CTX-I), a marker for bone resorption, in the serum of both 2-month-old and 8-month-old mice (Fig. 2I). RT-qPCR demonstrated that the mRNA expression of osteolytic markers, including *Trap*, *Nfatc1*, *cathepsin k*, and *DC-STAMP*, was significantly increased in femoral bone samples from 5-month-old *Usp26*^−/−^ mice compared with WT controls (Fig. 2J). Moreover, TRAP staining revealed the presence of an increasing number of osteoclasts in both the cortical and cancellous bone of *Usp26*^−/−^ mice (Fig. 2K, L). Collectively, these results demonstrate that *Usp26* deficiency leads to increased bone resorption.

### *Usp26* deficiency impairs osteoblastic differentiation of MSCs by decreasing β-catenin

**Fig. 3:**
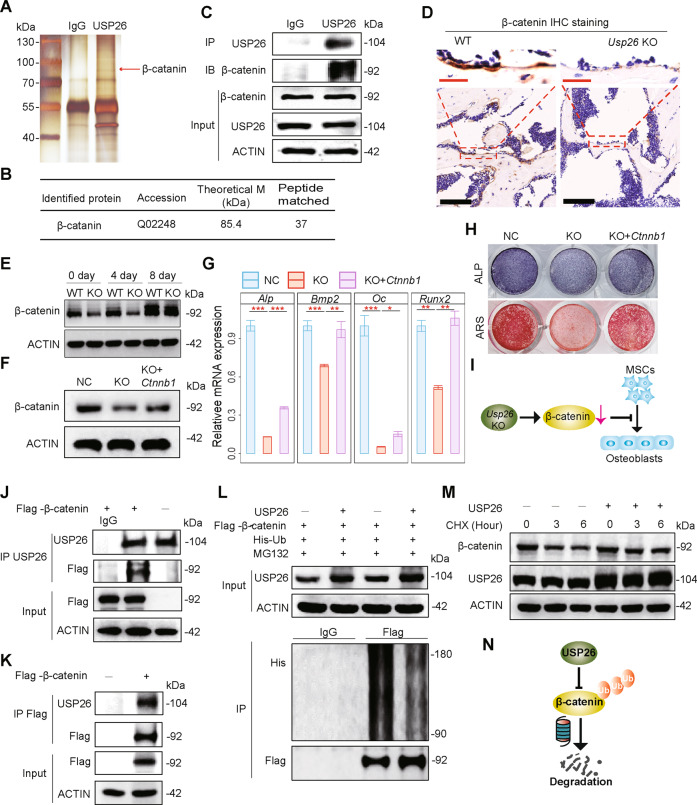
*Usp26* deficiency impairs osteoblastic differentiation of MSCs by decreasing β-catenin. **A** Silver stained SDS-PAGE image of USP26-binding proteins pulled down by USP26 antibody in the supernatant of MSCs lysates. **B** The accession number, theoretical molecular mass, and the number of non-redundant peptides of β-catenin identified by liquid chromatography–tandem mass spectrometry. **C** Co-immunoprecipitation of USP26 with endogenous β-catenin in mouse MSCs. **D** Representative of β-catenin IHC staining in femurs of 5-month-old *Usp26*^−/−^ mice and their WT littermates (*n* = 5). Red and black bars represent 25 and 100 μm, respectively. **E** Western blot analysis of β-catenin in MSCs obtained from *Usp26*^−/−^ and littermate controls with or without osteoblastic differentiation. **F** Western blot analysis of β-catenin in *Usp26*^−/−^ MSCs with or without *Ctnnb1* overexpression. **G**, **H** Osteoblastic genes expression, ARS and ALP staining of *Usp26*^−/−^ MSCs with or without *Ctnnb1* overexpression. **I** The schematic graph reflects that decreased protein of β-catenin was responsible for impaired osteoblastic differentiation of *Usp26*^−/−^ MSCs. **J**, **K** Co-immunoprecipitation of USP26 with ectopically expressed Flag-tagged β-catenin in 293 T cells. **L** Overexpression of *Usp26* decreases the level of ubiquitinated β-catenin. **M** Western blot analysis of the protein level of β-catenin in 293 T cells with or without *Usp26* overexpression and treated with cycloheximide (CHX) for indicated time intervals. **N** The schematic graph reflects the underlying mechanisms of USP26 in decreasing β-catenin degradation by reducing the level of ubiquitinated β-catenin in 293 T cells. *P < 0.05, **P < 0.01, ***P < 0.001. *P* values were analyzed by one-way ANOVA. All data are representative of two to three independent experiments.

As a deubiquitylating enzyme, USP26 is involved in cell differentiation by stabilizing several targets [13]. To explore the potential substrate involved in USP26-mediated osteoblastic differentiation of MSCs, we first performed protein pull-down assays and liquid chromatography–tandem mass spectrometry analysis to screen USP26-interacting proteins in MSCs. We found that β-catenin was detectable in USP26-binding proteins (Fig. 3A, B and Fig. S4). Further co-immunoprecipitation (Co-IP) assays demonstrated the enrichment of β-catenin in complexes precipitated with antibody against USP26 compared with control IgG (Fig. 3C). Since the β-catenin pathway plays a vital role in osteoblastogenesis [14], we investigated whether USP26 regulated the osteoblastic differentiation of MSCs via β-catenin. Reduced β-catenin protein was observed in the osteoblasts of femur samples from *Usp*26^−/−^ mice (Fig. 3D). In addition, a significant decrease in the protein abundance of β-catenin was also detected in *Usp26*^−/−^ MSCs, either at the basal level or after osteoblastic differentiation (Fig. 3E), whereas overexpression of *Ctnnb1*, a gene encoding β-catenin, significantly improved osteoblastic differentiation of MSCs, which had been impaired as a result of *Usp26* deficiency (Fig. 3F–H). These results indicated that decreased protein of β-catenin was responsible for the impaired osteoblastic differentiation observed in *Usp26*^−/−^ MSCs (Fig. 3I).

To test whether USP26 could regulate β-catenin degradation, we first ectopically expressed Flag-tagged β-catenin in 293 T cells and found that β-catenin was detected in USP26 immunoprecipitates and vice versa (Fig. 3J, K). Overexpression of USP26 significantly decreased the level of ubiquitinated β-catenin (Fig. 3L) and resulted in much slower degradation of β-catenin protein in the presence of cycloheximide, an inhibitor of protein translation (Fig. 3M). Collectively, these data indicated that USP26 could decrease β-catenin degradation in 293 T cells by reducing the level of ubiquitinated β-catenin (Fig. 3N).

### *Usp26* deficiency facilitates osteoclastic differentiation of BMMs by decreasing IκBα

**Fig. 4:**
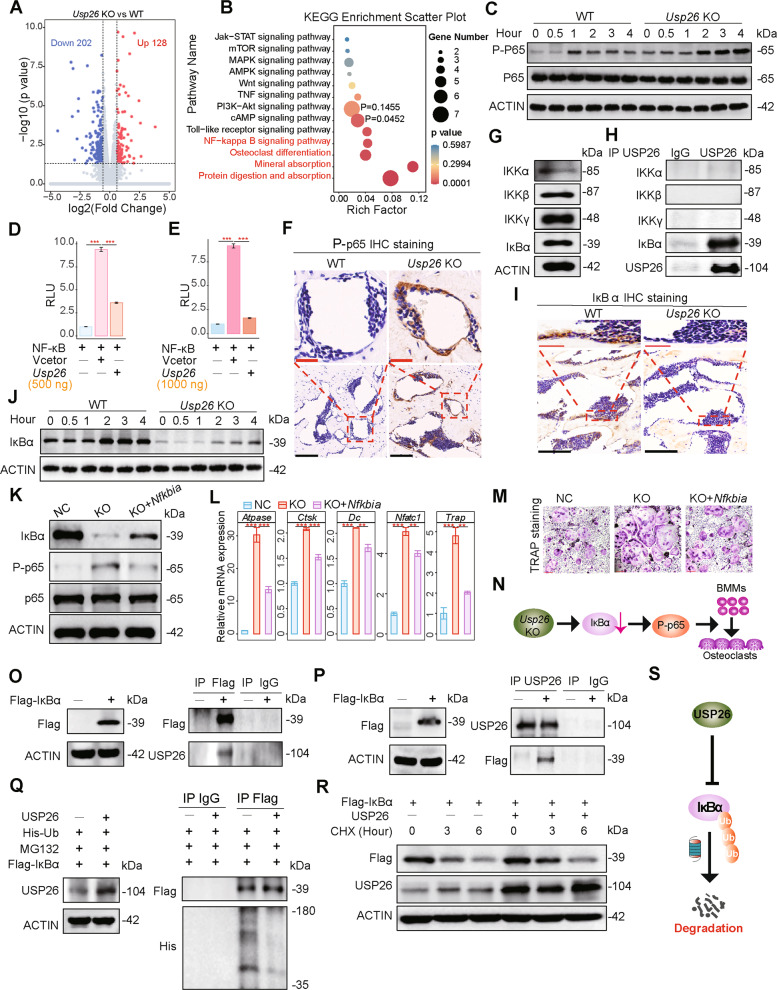
*Usp26* deficiency facilitates osteoclastic differentiation of BMMs by decreasing IκBα. **A** Volcano plots of differentially expressed genes in *Usp26*^−/−^ osteoclasts (*Usp26* KO) as compared with WT controls (WT). The blue and red dots represent the down- and upregulated genes, respectively. **B** KEGG enrichment analysis of the correlated signal pathway to the differently expressed mRNAs between *Usp26*^−/−^ and WT osteoclasts. **C** Western blot analysis of P-p65 in BMMs obtained from *Usp26*^−/−^ mice and littermate controls with or without osteoclastic differentiation. **D**, **E** NF-κB transcriptional activity in BMMs induced by osteoclastic differentiation with or without different doses of *Usp26* overexpression. **F** Representative of P-p65 IHC staining in femurs of 5-month-old *Usp26*^−/−^ mice and their WT littermates (*n* = 5). Red and black bars represent 25 and 100 μm, respectively. **G** Western blot analysis of IKKα, IKKβ, IKKγ, and IκBα expression in mouse BMMs. **H** Co-immunoprecipitation of USP26 with endogenous IKKα, IKKβ, IKKγ, and IκBα in mouse BMMs. **I** Representative of IκBα IHC staining in femurs of 5-month-old *Usp26*^−/−^ mice and their WT littermates (*n* = 5). Red and black bars represent 25 and 100 μm, respectively. **J** Western blot analysis of IκBα expression in BMMs isolated from *Usp26*^−/−^ mice and their WT littermates with or without osteoclastic differentiation. **K** Western blot analysis of IκBα and P-p65 expression in *Usp26*^−/−^ BMMs with or without *Nfkbia* overexpression. **L**, **M** The osteoclastic genes expression and TRAP staining of *Usp26*^−/−^ osteoclasts with or without *Nfkbia* overexpression. **N** The schematic graph reflects that decreased IκBα results in increased NF-κB activation and enhanced osteoclastic differentiation in *Usp26*^−/−^ BMMs. **O**, **P** Co-immunoprecipitation of USP26 with ectopically expressed Flag-tagged IκBα in 293 T cells. **Q** Overexpression of USP26 decreases the level of ubiquitinated IκBα. **R** Western blot analysis of ectopically expressed IκBα protein in 293 T cells with or without USP26 overexpression and treated with cycloheximide (CHX) for indicated time intervals. **S** The schematic graph reflects the underlying mechanisms of USP26 in decreasing IκBα degradation by reducing the level of ubiquitinated IκBα in 293 T cells. **P < 0.01, ***P < 0.001. *P* values were analyzed by one-way ANOVA. All data are representative of two to three independent experiments.

To detect the underlying mechanisms by which USP26 regulates osteoclastic differentiation of BMMs, the whole transcriptome of WT and *Usp26*^−/−^ osteoclastic cells was analyzed. Among the significantly and differentially expressed transcripts, 202 genes were downregulated (<0.66-fold, *p* < 0.05), and 128 genes were upregulated (>1.5-fold, *p* < 0.05) in *Usp26*^−/−^ osteoclastic cells compared with WT controls (Fig. 4A). Kyoto Encyclopedia of Genes and Genomes (KEGG) enrichment analysis revealed that in addition to being associated with signaling pathways concerning protein digestion, minimal absorption, and osteoclast formation, NF-κB was strongly correlated with differentially expressed mRNAs between WT and *Usp26*^−/−^ osteoclasts (Fig. 4B).

NF-κB activation is pivotal in osteoclastogenesis [15]; therefore, we sought to test whether USP26 regulated osteoclastic differentiation of BMMs by regulating NF-κB activation. Our results showed that *Usp26* deficiency significantly increased the phosphorylated p65 component (P-p65) of NF-κB signaling, both at the basal level and after osteoclastic differentiation (Fig. 4C), while *Usp26* overexpression significantly inhibited NF-κB transcriptional activity (Fig. 4D, E). In addition, a significant increase in P**-**p65 was observed in BMMs and the osteoclasts of femur tissues from *Usp26*^−/−^ mice compared with WT controls (Fig. 4F). Therefore, *Usp26* deficiency may facilitate osteoclastic differentiation of BMMs by increasing NF-κB-p65 activation.

Despite the diversity of upstream stimuli, the NF-κB cascade shares a common activation scheme consisting of activation of the IκB-kinase complex (IKK), composed of a catalytic subunit (IKKα or IKKβ) and a regulatory subunit (IKKγ), followed by the phosphorylation, ubiquitination, and degradation of IκB (inhibitors of NF-κB) proteins, which results in p65 phosphorylation and NF-κB activation [16]. Here, we found that IKKα, IKKβ, IKKγ, and IκBα were all detectable in preosteoclasts (Fig. 4G). Therefore, to detect the upstream adapters involved in NF-κB activation regulated by USP26, USP26 immunoprecipitates were immunoblotted with specific antibodies against IKKα, IKKβ, IKKγ and IκBα. The results showed that only IκBα could be detected (Fig. 4H). Meanwhile, reduced levels of IκBα protein were detected in the osteoclasts of femur samples of *Usp26*^−/−^ mice compared with those of WT controls (Fig. 4I). Furthermore, *Usp26* deficiency significantly decreased the protein level of IκBα, either at the basal level or after osteoclastic differentiation (Fig. 4J). Gain-of-function experiments revealed that overexpression of *Nfkbia*, a gene encoding IκBα, decreased P-p65, inhibited osteoclastic gene expression, and impaired multinucleated osteoclast formation in *Usp26*^−/−^ BMMs (Fig. 4K–M). Collectively, these results demonstrated that *Usp26* deletion in BMMs resulted in decreased IκBα, which was responsible for increased NF-κB activation and osteoclastic differentiation (Fig. 4N).

To investigate whether USP26 mediates the regulation of IκBα protein degradation, Flag-tagged IκBα was ectopically expressed in 293 T cells, and Co-IP assays were performed. Notably, USP26 was detectable in anti-Flag immunoprecipitates and vice versa (Fig. 4O, P). Overexpression of USP26 significantly decreased the level of ubiquitinated IκBα (Fig. 4Q) and resulted in much slower degradation of IκBα protein in the presence of cycloheximide (Fig. 4R). Taken together, these results demonstrated that USP26 decreased IκBα degradation in 293 T cells via its deubiquitylating activity (Fig. 4S).

### *Usp26* facilitates bone regeneration

**Fig. 5:**
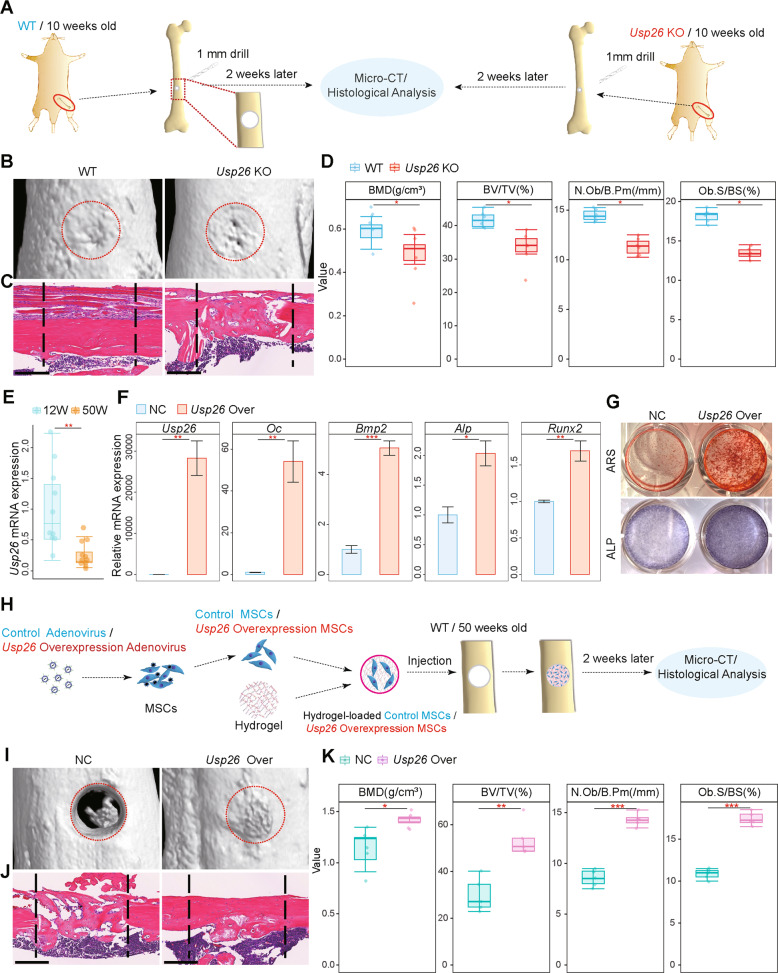
*Usp26* facilitates bone regeneration. **A** 1.0 mm holes were generated in femoral bones of 10-weeks old *Usp26*^−/−^ male mice and their littermate controls, and the defect bone samples were collected for micro-CT scanning and histology analysis 2 weeks after the surgery. **B** Representative micro-CT images of femoral cortical bone defects in 10-week-old WT and *Usp26*^−/−^ male littermates (*n* = 6). The red dotted lines indicate the position of the original defect margin. **C** H&E staining of femoral cortical bone defects (*n* = 6). The black dotted lines indicate the position of the original defect margin. Scale bars represent 200 μm. **D** Bone volume (BV/TV, %), bone mineral density (BMD, g/cm^3^), osteoblast numbers (N.Ob/B.Pm(/mm)), and osteoblast surface (Ob.S/BS (%)) of the regenerated bone in femoral cortical gaps (*n* = 6). **E** Quantification analysis of *Usp26* expression in MSCs isolated from 12-week-old and 50-week-old mice (*n* = 10). **F** Quantification analysis of *Usp26*, *Oc*, *Bmp2*, *Alp*, and *Runx2* expression in MSCs isolated from aged mice after 8 days of osteoblastic differentiation with or without *Usp26* overexpression (*n* = 5). **G** Representative images of ARS and ALP staining in MSCs isolated from aged mice after 8 days of osteoblastic differentiation with or without *Usp26* overexpression (*n* = 3). **H** 1.0 mm holes were generated in femoral bones of 50-weeks old male mice, then hyaluronic acid hydrogel containing *Usp26*-overexpressed MSCs or the control MSCs were injected into the bone defects. After 2 weeks of recovery, femoral bones were collected and newly formed bones were analyzed by reconstructing 3D micro-CT images and histology analysis. **I** Representative micro-CT images of femoral cortical bone defects in 50-week-old mice with or without *Usp26*-overexpressed MSCs injection (*n* = 5). The red dotted lines indicate the position of the original defect margin. **J** H&E staining of femoral cortical bone defects in 50-week-old mice with or without *Usp26*-overexpressed MSCs injection (*n* = 5). Scale bars represent 200 μm. **K** Bone volume (BV/TV, %), bone mineral density (BMD, g/cm^3^), osteoblast numbers (N.Ob/B.Pm(/mm)), and osteoblast surface (Ob.S/BS (%)) of the regenerated bone in femoral cortical gaps of 50-week-old mice with or without *Usp26*-overexpressed MSCs injection (*n* = 5). *P < 0.05, **P < 0.01, ***P < 0.001. *P* values were analyzed by two-tailed *t* tests. All data are representative of two to three independent experiments.

Osteoblastic differentiation of MSCs is important for bone defect regeneration [17]. We surgically created skeletal defects by drilling holes in femoral cortical bone to evaluate the effect of USP26 on bone regeneration (Fig. 5A). Micro-CT and histological analysis consistently showed that the cortical gaps in WT mice were almost completely bridged after 2 weeks, whereas those in *Usp26*^−/−^ mice were only partially filled (Fig. 5B, C). In addition, the BV and BMD of the mineralized calli of *Usp26*^−/−^ mice were significantly lower than those of their WT littermate controls (Fig. 5D). The osteoblast numbers and osteoblast surfaces in regenerated bone of *Usp26*^−/−^ mice were also diminished compared with those of WT controls (Fig. 5D). This indicated that USP26 is essential for bone regeneration.

Age-related decline in the osteoblast potential of MSCs is one of the pivotal triggers for impaired bone defect healing in elderly people [18]. Our results revealed that *Usp26* expression in MSCs was inversely correlated with aging in mice (Fig. 5E). Furthermore, *Usp26* overexpression significantly increased the osteoblastic differentiation activity of MSCs isolated from aged mice (Fig. 5F, G). To determine the potential for clinical application of USP26 in bone regeneration in elderly mice, a hyaluronic acid hydrogel containing *Usp26*-overexpressing MSCs or a control was injected into the bone defects of 50-week-old mice. After 2 weeks of recovery, newly formed bones were analyzed by micro-CT and histological analysis (Fig. 5H). MSCs with *Usp26* overexpression had significantly increased regenerated BV (Fig. 5I–K). Histological examination further confirmed the increased bone formation, osteoblast numbers and osteoblast surfaces at the defect margins (Fig. 5K). Taken together, these data demonstrate that USP26 facilitates bone regeneration.

### *Usp26* supplementation decreases bone loss induced by ovariectomy

**Fig. 6:**
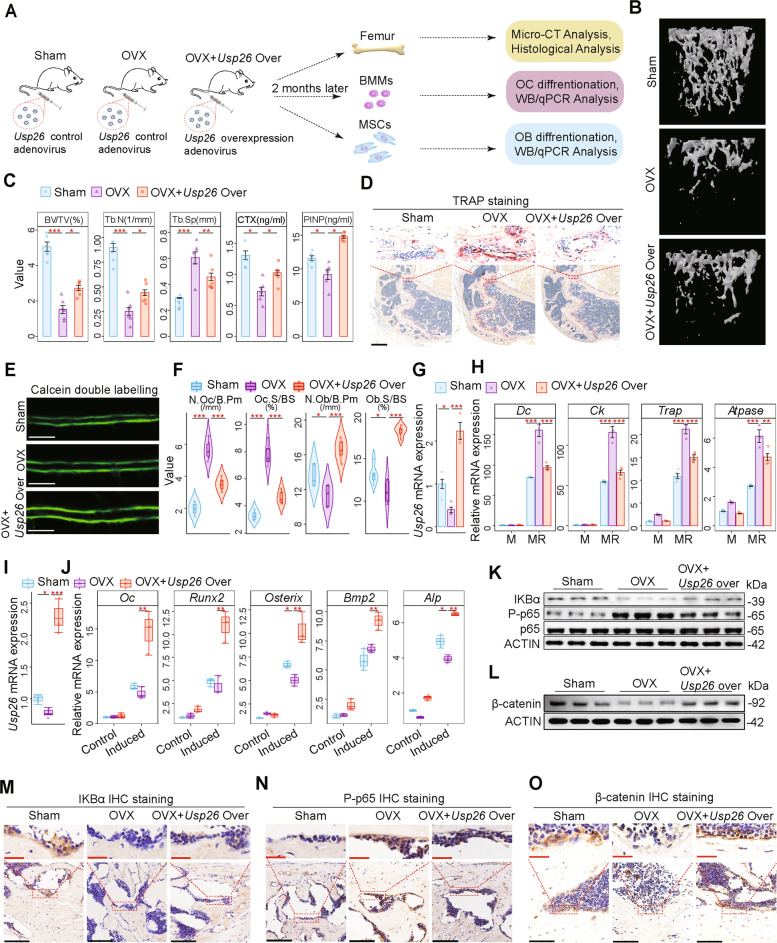
*Usp26* supplementation decreases the bone loss induced by ovariectomy. **A** Ovariectomized mice were treated with intravenous injection of *Usp26* overexpression adenovirus or control adenovirus for 2 months. Then the mice were euthanized and randomly assigned for femur bone micro-CT and histological analysis, MSCs and BMMs isolation. **B** Representative micro-CT images of trabecular bone from the femoral metaphysis of the sham-operated (Sham), ovariectomized (OVX) and *Usp26* overexpression adenovirus-treated OVX mice (OVX + *Usp26* Over). **C** Cancellous bone volume (BV/TV, %), trabecular number (Tb.N, 1/mm), and trabecular separation (Tb.Sp, mm) were determined by micro-CT analysis, CTX-I and PINP concentration in serum were detected with ELISA (*n* = 6). **D** TRAP staining of femurs of Sham, OVX, and OVX + *Usp26* Over mice (*n* = 6). Scale bars represent 200 μm. **E** Calcein double labeling in femurs of Sham, OVX, and OVX + *Usp26* Over mice (*n* = 3). Scale bars represent 200 μm. **F** Parameters for osteoclasts and osteoblasts in the bone morphometric analysis of Sham, OVX, and OVX + *Usp26* Over mice (*n* = 6). **G**, **H** Quantification of *Usp26*, *Dc* (*DC-STAMP*), *Ck* (*cathepsin K*), *Trap*, and *Atpase* (*V-ATPase α3*) expression in BMMs isolated from Sham, OVX, and OVX + *Usp26* Over mice after 3 days of osteoclastic differentiation (*n* = 3 or 4). **I**, **J** Quantification of *Usp26*, Oc, *Runx2*, osterix, *Bmp2*, and *Alp* expression in MSCs isolated from Sham, OVX, and OVX + *Usp26* Over mice after 8 days of osteoblastic differentiation (*n* = 3). **K**, **L** Western blot analysis of IκBα and P-p65 in BMMs, β-catenin in MSCs isolated from Sham, OVX, and OVX + *Usp26* Over mice (*n* = 3). **M**–**O** IκBα, P-p65, and β-catenin IHC staining in femurs of Sham, OVX, and OVX + *Usp26* Over mice (*n* = 4). Red and black bars represent 25 and 100 μm, respectively. *P < 0.05, **P < 0.01, ***P < 0.001. *P* values were analyzed by one-way ANOVA. All data are representative of two to three independent experiments.

To evaluate the potential role of USP26 in bone loss induced by ovariectomy, ovariectomized 10-week-old mice were treated with intravenous injection of *Usp26* overexpression adenovirus or a control starting 2 days after ovariectomy (Fig. 6A). Micro-CT analysis revealed that *Usp26* overexpression decreased bone loss induced by ovariectomy (Fig. 6B, C). Accordingly, the serum concentrations of CTX-I and PINP indicated decreased osteoclastic activity and increased osteoblastic activity (Fig. 6C). In addition, impaired osteoclastic bone resorption and advanced osteoblastic bone formation after *Usp26* overexpression were further confirmed by TRAP staining and calcein double-labeling analysis (Fig. 6D, E). Consistently, osteoclast numbers and osteoclast surfaces significantly decreased, whereas osteoblast numbers and osteoblast surfaces significantly increased after *Usp26* overexpression (Fig. 6F). To evaluate the effect of *Usp26* supplementation on the osteoclastic differentiation of BMMs and osteoblastic differentiation of MSCs, BMMs and MSCs were isolated from sham-operated (Sham), ovariectomized (OVX), and *Usp26* overexpression adenovirus-treated OVX mice (OVX + *Usp26* Over), and their osteoclastic and osteoblastic differentiation activities were compared. The results showed that *Usp26* expression was decreased in both the BMMs and MSCs of OVX mice (Fig. 6G, I), and *Usp26* overexpression not only dampened the hyperactivity of osteoclastic differentiation of BMMs (Fig. 6G, H) but also advanced the osteoblastic differentiation of MSCs after ovariectomy (Fig. 6I, J). More importantly, *Usp26* overexpression increased IκBα and decreased P-p65 in ovariectomized BMMs and increased β-catenin in ovariectomized MSCs (Fig. 6K, L). Moreover, the same effect of USP26 overexpression on IκBα, P-p65, and β-catenin was found in the osteoclasts and osteoblasts of femur samples of ovariectomized mice, as evidenced by immunohistochemical staining analysis (Fig. 6M–O). Taken together, these results demonstrate that *Usp26* supplementation decreases bone loss induced by ovariectomy.

## Discussion

Bone integrity is maintained by bone-forming osteoblasts and bone-resorbing osteoclasts [5]. Identifying the molecules that regulate both bone formation and resorption simultaneously is essential for designing improved therapeutics for treating metabolic bone diseases such as osteoporosis. In the present study, we identified USP26 as a previously unknown regulator of bone homeostasis that simultaneously alters bone formation and resorption.

USP26 was first identified by Wang et al., who isolated this gene from mouse spermatogonia [19]. A human homolog that displays a testis-specific expression pattern has also been identified [20]. Several studies reported the presence of different polymorphisms in USP26 in patients with nonobstructive azoospermia or severe oligozoospermia, suggesting that alterations in USP26 might be involved in male infertility [21, 22]. In addition, USP26 has also been recognized as a regulator of the androgen receptor hormone-induced action that is involved in spermatogenesis and steroid production [23]. However, the involvement of USP26 in subfertility has yielded controversial results, and it was found that the impaired fertility and spermatogenesis caused by *Usp26* mutations in mice were dependent on genetic background [24]. In line with this study, we found that *Usp26* heterozygotes were viable and born at the expected Mendelian ratio, and mutant males backcrossed to a C57BL/6 background showed normal reproductive ability.

Further in-depth studies also showed that *Usp26* was expressed in other organs. In addition, USP26 promotes esophageal squamous cell carcinoma metastasis by stabilizing Snail [25]. USP26 was also found to be requisite for embryonic stem cell differentiation because it stabilizes PRC1 complex components [26]. However, whether USP26 participates in osteoblastic differentiation of MSCs or osteoclastic differentiation of BMMs is unknown. Here, we provide the first evidence that *Usp26* is expressed in MSCs and BMMs. We also uncovered a vital role of *Usp26* in regulating bone homeostasis: *Usp26* facilitates the osteoblast differentiation of MSCs and antagonizes the osteoclastic differentiation of BMMs. Mechanistically, USP26 stabilizes β-catenin to promote the osteogenic activity of MSCs and impairs the osteoclastic differentiation of BMMs by stabilizing IκBα.

In light of the dual role of *Usp26* in facilitating osteoblastic differentiation and antagonizing osteoclastic differentiation, we studied the potential for preclinical application of *Usp26* in mouse models of aged bone defect regeneration and ovariectomized bone loss. Interestingly, we found that *Usp26* expression was decreased in aged MSCs, BMMs and MSCs isolated from ovariectomized mice. Since bone loss in ovariectomized mice is largely due to estrogen deficiency, we found that estrogen induced *Usp26* expression in BMMs and MSCs (Fig. S7). Multiple studies suggest that age-induced oxidative stress may contribute to osteoporotic bone loss and impaired bone defect healing by inhibiting osteoblastic differentiation of MSCs [27–29]. We found that oxidative stress significantly inhibited *Usp26* expression in MSCs (Fig. S6). These preliminary data indicated that aging and estrogen deficiency regulate USP26 expression.

Although our present study clearly demonstrated impaired osteoblastic differentiation of *Usp26*^−/−^ MSCs, it must be noted that MSCs are also precursors of chondrocytes [30, 31]. Abnormal differentiation of MSCs into chondrocytes results in chondrocyte defects, disturbs early skeletal development, and leads to an osteopenic phenotype [32, 33]. We found that *Usp26* deficiency dampened the chondrogenesis of MSCs, leading to a dramatic decrease in chondrocyte density in femur sections from embryonic day 16.5 (E16.5) *Usp26*^−/−^ mice (Fig. S5C, D). Skeleton Alizarin red and Alcian blue costaining results revealed that neither the calvaria, forelimb, nor hind limb of *Usp26*^−/−^ mice developed as well as that of the littermate controls (Fig. S5E–H). These preliminary results revealed that *Usp26* deletion impaired chondrogenesis of MSCs, resulting in decreased chondrocyte formation and abnormal early skeletal development.

Functional cross-talk between osteoblasts and osteoclasts plays a key role in maintaining bone homeostasis [34]. Osteoblasts regulate the differentiation and maturation of osteoclasts by producing several factors [35, 36]. *Runx2* in osteoblasts promotes osteoclast differentiation by inducing *Rankl* [37]. Glass et al. demonstrated that the β-catenin pathway in differentiated osteoblasts inhibits osteoclast differentiation by driving the expression of *Opg*, a gene encoding a decoy receptor for RANKL [38]. Herein, we found that *Usp26*^−/−^ mice show decreased bone formation and increased bone resorption, and *Usp26* deficiency decreased the protein levels of β-catenin and *Runx2* expression in MSC-derived osteoblastic cells. Therefore, it is reasonable to speculate that *Usp26* in osteoblast lineage cells, such as differentiated osteoblasts, could potentially control osteoclastic activity by regulating the expression of *Opg* and *Rankl*. Disruption of *Usp26* expression in different osteoblast lineage cells is needed to better understand the role of USP26 in osteoclastogenesis.

Taken together, our data demonstrate that USP26 coordinates bone formation and resorption by facilitating osteoblastic differentiation via β-catenin and antagonizing osteoclastic differentiation via IκBα. Furthermore, the identification of USP26 in driving bone regeneration and protecting against OVX-induced bone loss indicates that USP26 represents a potential therapeutic target for metabolic bone diseases.

## Materials and methods

### Mice

*Usp26*^+/−^ C57BL/6 breeding pairs (Stock No: T001875) were purchased from GemPharmatech Co, Ltd (Nanjing, Jiangsu province, China). *Usp26*^−/−^ mice and their littermate controls were obtained by the crossbreeding of *Usp26*^+/−^ mice. C57BL/6 were purchased from Shanghai Laboratorial Animal Center, Chinese Academy of Sciences (Shanghai, China). The animals were housed with free access to water and diet in an air-conditioned room with a 12-h light-dark cycle, at 21–23 °C and 60% relative humidity in the animal facility at Ruijin Hospital, Shanghai Jiao Tong University (SJTU) School of Medicine, Shanghai, China. For all mouse studies, we performed preliminary experiments to determine requirements for sample size. Mice were assigned randomly to experimental groups but not performed in a blinded manner.

### Ethics statement

All animal experiments were performed according to the protocol approved by the SJTU Animal Care and Use Committee and in direct accordance with Ministry of Science and Technology of the People’s Republic of China on Animal Care guidelines [IACUC protocol number: SYXK (Shanghai) 2018-0027]. All surgeries were performed under anesthesia and all efforts were made to minimize suffering.

### Cell culture

Tibiae and femurs of 8-week-old *Usp26*^−/−^ mice and their WT littermate controls were used for primary MSCs and BMMs isolation by flushing the bone marrow with α-MEM [36, 39]. Cells were seeded on 100-mm culture dishes (Nunc, Rochester, NY), and cultured in α-MEM supplemented with 100 IU/ml penicillin, 100 mg/ml streptomycin (Gibco BRL), and 10% fetal bovine serum (Gibco BRL). After 16 h of culture, the adherent and non-adherent cells were harvested for subsequently MSCs and BMMs culture. To induce osteogenic differentiation, MSCs were seeded in 6-well or 24-well plates. At 80% confluence, cells were treated with osteogenic medium containing 50 μM ascorbic acid (Sigma), 10 mM β-glycerophosphate (Sigma), and 10 nM dexamethasone (Sigma). To induce osteoclastic differentiation, BMMs were seeded in 24-well plates supplemented with 50 ng/ml M-CSF for 3 days. Adherent cells were harvested as osteoclast progenitors and were further cultured with α-MEM containing M-CSF (30 ng/ml) and RANKL (50 ng/ml) for another 3–5 days.

### Quantitative real-time RT-PCR

Trizol reagent (Invitrogen, Carlsbad, CA, USA) was used for total RNA extraction. For reverse transcription of mRNA, 1 μg of total RNA was used for reverse transcription with Prime-Script RT reagent kit (#RR036A, Takara Biotechnology, Japan). Quantitative real-time PCR was performed to amplify the cDNA by the SYBR Premix Ex Tag kit (Takara Biotechnology, Japan) and ABI 7500 Sequencing Detection System (Applied Biosystems, Foster City, CA, USA). β-actin was used as endogenous control for quantitation of mRNA expression. The specific primer sequences for real-time RT-PCR were described in Table S1 in Supplementary Materials. The samples with low yield of RNA were predetermined and excluded.

### Bone histomorphometry and immunohistochemistry

Bone tissues were embedded with paraffin after decalcification. Five μm-thick sections were stained with H&E and TRAP staining according to standard methods, respectively. The ratio of osteoblast/osteoclast numbers to bone perimeter (N.Ob/B.Pm (/mm)), (N.Oc/B.Pm (/mm)) and the ratio of osteoblast/osteoclast surface to bone surface (Ob.S/BS (%)), (Oc.S/BS (%)) were quantified with software (OsteoMetrics, Decatur, GA) on H&E-stained and TRAP-stained sections at 200× magnification. All histomorphometric parameters were calculated and expressed according to the suggestions made by the ASBMR nomenclature committee [40].

Processing of undecalcified bone specimens and cancellous bone histomorphometry was performed as described previously [1, 41]. Femurs were dehydrated and embedded in methyl methacrylate. 5μm-thick sections were prepared using a Leica RM2235 microtome and were stained by the von Kossa/nuclear fast red method.

Fluorochrome double labeling was performed as previously report [42]. A double calcein (25 mg/kg) label was injected subcutaneously at 10 and 3 days before necropsy. Non-decalcified bone specimens of femurs were made and the double calcein label was by means of a microscope (Olympus, Japan) equipped with a digital camera (Olympus, Japan).

For IHC analysis, deparaffinized sections were incubated with 3% H_2_O_2_ for 15 min, and then treated with 5% BSA for 10 min. Next, the sections were incubated with BMP2 (ab14933, Abcam), osteocalcin (ab93876, Abcam), osterix (ab22552, Abcam), β-catenin (#8480, CST), P-p65 (ab86299, Abcam) and IκBα (#4814, CST) primary antibodies overnight at 4 °C, respectively. Followed by incubated with biotin conjugated secondary antibodies, and visualized with the streptavidin-biotin staining technique. Nucleus was stained with hematoxylin and the slides were photographed by a microscope (ZEISS, AXIO). For all staining, inadequate staining samples due to technical problems were excluded.

### Western blot and Co-IP

Cell samples were lysed in RIPA buffer on ice for 20 min. The lysed cells were collected and centrifuged at 14,000 × *g* to remove cell debris. Protein concentrations were determined with the BCA Protein Assay Kit (P0009, Beyotime). The samples with low yield of protein were predetermined and excluded. Each sample containing 10 μg of total protein was separated by SDS-PAGE in a 10% gel and transferred onto PVDF membranes (EMD Millipore Corporation, US). After blockage with 5% nonfat dry milk in Tris-buffered saline with 1‰ Tween (TBST), the membranes were incubated overnight at 4 °C with primary antibodies against osterix (ab22552, Abcam), RUNX2 (ab23981, Abcam), USP26 (ab230226, Abcam), β-catenin (#8480, CST), β-actin (#58169 S, CST), Flag-Tag (T0053, Affinity), His-Tag (D291-3, MBL, Japan), p65 (#8242 S, CST), P-p65 (AP0475, ABclonal, Wuhan, China), IKKα (#2682, CST), IKKβ (#8943, CST), IKKγ (ab178872, Abcam), IκBα (#4814, CST). After three washes with TBST, the membrane was incubated with horseradish peroxidase–conjugated secondary antibodies (Jackson). The antibody–antigen complexes were visualized with Immobilon reagents (Millipore).

For Co-IP, whole-cell extracts were prepared by using lysis buffer after transfection or stimulation and were incubated with the appropriated antibodies overnight at 4 °C. Protein A&G beads (Abmart) were added and the incubation was continued for 4 h at 4 °C. Coprecipitated proteins were washed, eluted with SDS-loading buffer at 95 °C for 5 min, and then subjected to western blot analyses.

### Surgery for femoral cortical defect

Surgery of femoral cortical bone defect was performed as previous report [1]. Ten-week-old *Usp26*^−/−^ male mice and the littermate controls were anesthetized by intraperitoneal injection of pentobarbital sodium. A 5-mm longitudinal incision was made over the middle femur and the bone surface was exposed by splitting the muscle. A 1.0 mm hole was generated using a round bur (Komet^®^, Germany) operating at 10,000 rpm under saline irrigation. Samples were collected for micro-CT scanning and histology analysis 2 weeks after the surgery.

To evaluate *Usp26*-overexpressed MSCs in femoral cortical defect in elders, 50-week-old C57BL/6 male mice were anesthetized by intraperitoneal injection of pentobarbital sodium. A 5-mm longitudinal incision was made over the middle femur and the bone surface was exposed by splitting the muscle. A 1.0 mm hole was generated using a round bur (Komet^®^, Germany) operating at 10,000 rpm under saline irrigation. Then hyaluronic acid hydrogel containing *Usp26*-overexpressed MSCs or the controls were injected into the bone defects. After 2 weeks of recovery, samples were collected and newly formed bones were analyzed by reconstructing 3D micro-CT images and histology analysis.

### Ovariectomy-induced bone loss

Ten-week-old female mice were ovariectomized or sham-operated. Ovariectomized mice were treated with intravenous injection of *Usp26* overexpression adenovirus or control adenovirus starting 2 days after ovariectomy. All the mice were euthanized after 2 months later, three mice of each group were randomly assigned for MSCs and BMMs isolation and subsequently for osteoblastic differentiation and osteoclastic differentiation, respectively. The samples of the resting 7 mice in each group were collected for micro-CT scanning and histology analysis.

### Micro-computed tomography

Micro-CT analysis was performed on the left femur of each mouse as described previously [43]. After fixation with 4% paraformaldehyde, the femurs were scanned on a Skyscan 1172 (Aartselaar, Belgium) with a 10-μm isotropic voxel size, 50 keV, 500 μA, and 0.7° rotation step, in accordance with the recommendations of the American Society for Bone and Mineral Research (ASBMR) [40]. Regions of interest (ROIs) were defined for trabecular and cortical parameters. The trabecular ROI extended from 1 mm proximally to the end of the distal growth plate over 1 mm toward the diaphysis. The cortical ROI extended from 3 mm proximally to the end of the distal growth plate over 1 mm toward the diaphysis. The resulting two-dimensional images of trabecular and cortical bone in relative cross-sections were shown in grayscale. Trabecular bone parameters were measured including BMD (g/cm^3^), BV/TV (%), trabecular thickness (Tb.Th, mm), and trabecular separation (Tb.Sp, mm). Cortical bone parameters were measured including BMD (g/cm^3^), total cross-sectional cortical bone area (B. Ar, mm^2^), cortical thickness (Ct. Th, mm) and Cortical porosity (Ct.Po, %).

For the analysis of cortical bone regeneration, the volume of interest (VOI) was defined as a cylindrical area covering the initial bone defect. BV/TV and BMD were calculated within the delimited VOI.

### Three-point bending test

The right femurs from all groups were immediately subjected to a three-point bending test with an Instron 5569 materials mechanical testing system (Instron Inc., MA) [43]. Femurs were placed posterior side down between two supports 6 mm apart, and load was applied at the midspan, which made bending occur along the anteroposterior axis. Load-displacement curves were recorded at a crosshead speed of 1 mm/s.

### ELISA

Concentrations of PINP and CTX-I in serum were determined using ELISA kits from IDS (Fountain Hills, AZ) according to the manufacturer’s instruction. The samples with low yield of protein were predetermined and excluded.

### Skeletal staining

Skeletal preparation and staining were performed as previous report [44]. One-week-old *Usp26*^−/−^ mice and the littermate controls were eviscerated and the skin was removed, and the resulting samples were transferred into acetone for 48 h after overnight fixation in 95% ethanol. After 3 days, the samples were rinsed with water and stained for 2 days with 0.005% ARS and 0.015% Alcian blue in ethanol. After rinsing with water, the samples were kept in 20% glycerol/1% KOH until the skeletons became clearly visible. For storage, they were serially transferred into 50%, 80%, and 100% glycerol.

### Lentivirus/small interfering RNA (siRNA preparation and targeting genes overexpression or knockdown

For gene overexpression, mouse *Ctnnb1*, *Usp26* or *Nfkbia* was cloned into a lentiviral vector backbone-pLV[Exp]-EGFP:T2A:Puro-EF1A via Golden Gate method [45], and mCherry was also inserted into the same vector backbone to make a negative control. For gene silencing, three different shRNA targeting mouse *Usp18*, *Usp21*, and one negative control shRNA (Table S2) were separately ligated into the lentiviral vector backbone-pLV[shRNA]-EGFP:T2A:Puro-U6 via restriction enzyme digestion and ligation. All vectors were validated by Sanger Sequencing and Restriction Enzyme Digestion Assay at the last step of vector construction.

To create lentivirus, each of the overexpression vectors or silencing vectors were cotransfected with pLV/helpr-SL3 (gag/pol element), pLV/helper-SL4 (pRev element) and pLV/helper-SL5 (pVSVG element), by calcium phosphate transfection method, into HEK293T cells. 48 h post transfection, supernatant containing the lentiviral particles was collected for concentrate and purify to make the final lentivirus for transduction, and the titers were confirmed by Lenti-X p24 Rapid Titer Kit.

siRNA oligonucleotides targeting *Usp4* (Table S3) were designed and synthesized by Genepharma (Shanghai, China). Overexpression lentivirus for each *Ctnnb1*, *Usp26* or *Nfkbia*, A mixture of three shRNA targeting *Usp18* or *Usp21*, or three siRNA oligonucleotides for *Usp4* or *Usp21* was used to transfected MSCs or BMMs. Overexpression or blockage efficiency was tested by either western blot or RT-qPCR.

### Luciferase reporter assay

Phosphorylated NF-κB-p65 (P-NF-κB-p65) promoter reporter with pGL6-Basic luciferase vector was purchased from Beyotime (Cat: D2206, Shanghai, China). Mouse BMMs were seeded into 24-well plates cotransfected with *Usp26* overexpression plasmid and firefly reporter constructs containing P-NF-κB-promoter reporter and Renilla-expressing plasmid for 24 h. Firefly and Renilla luciferase activities were measured 6 h after osteoclastic induction by a Dual Luciferase Assay System (Promega).

### Protein pull-down assays and liquid chromatography–tandem mass spectrometry assay

The supernatant of MSCs lysates were incubated with anti-USP26 antibody overnight at 4°C. After that the protein A + G beads were added to pull down the interacting proteins of USP26. The bound proteins were eluted from the packed beads and analyzed by SDS-PAGE. After examination of the silver staining, the gel bands containing USP26-binding protein were clipped out and cut into small pieces for liquid chromatography–tandem mass spectrometry (Easy Nlc1200/Q Exactive Plus). The sequences from mass spectrometry were further analyzed with Mascot and NCBI.

### The whole transcriptome analysis

RNA extraction, mRNA library construction and sequencing were performed as previous report [46]. After the final transcriptome was generated, StringTie and ballgown were used to estimate the expression levels of all transcripts and genes by calculating FPKM (FPKM = [total_exon_fragments/mapped_reads(millions) × exon_length(kB)]). The differentially expressed transcripts and genes were selected with fold change > 1.5 or fold change < 0.66 and *P* value < 0.05 by R package edgeR. The correlated signal pathways to the differently expressed mRNAs were enriched by KEGG pathway analysis. The raw data were submitted to NCBI BioProject database under accession number PRJNA763041.

### Statistical analysis

All data representative of three independent experiments are present as mean ± S.E.M. We used two-tailed *t* tests to determine significances between two groups. We did analyses of multiple groups by one- or two-way ANOVA with Bonferroni post-test of GraphPad prism version 5. For all statistical tests, we considered *P* value < 0.05 to be statistically significant.

## Supplementary information

Supplementary files

Reproducibility Checklist

Detailed Author Contribution form

## Data Availability

All data needed to evaluate the conclusions in the paper are present in the paper and/or the Supplementary Materials. Data related to this paper may be requested from the authors.
