# Supplementary material for: The osteoprotective role of USP26 in coordinating bone formation and resorption
Source: Cell Death Differ. 2022 Jan 29;29(6):1123–36. doi: 10.1038/s41418-021-00904-x (PMC9177963; doi:10.1038/s41418-021-00904-x)
Supplement: Supplementary file 1 — Supplementary files [file 41418_2021_904_MOESM1_ESM.pdf]

Supplementary files

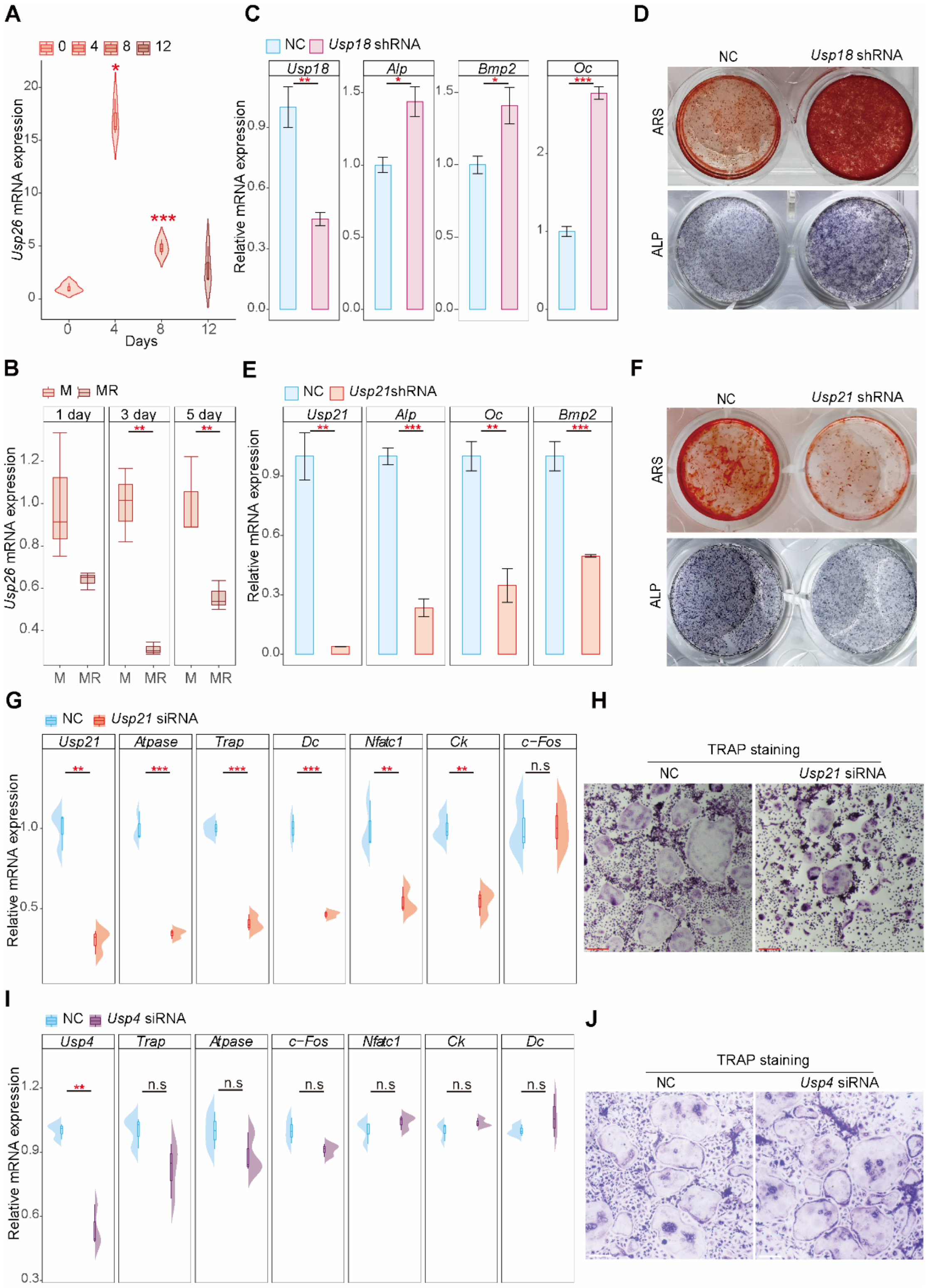

Fig. S1. ***Usp26*** **expression during osteoblastic and osteoclastic differentiation, and the effect of** ***Usp18*****,** ***Usp21*****, or** ***Usp4*** **deletion on**

osteoblastic or osteoclastic differentiation. (A) Time curve of *Usp26* expression in MSCs after different days of osteoblastic differentiation. (B) Time curve of *Usp26* expression in BMMs after different days of osteoclastic differentiation. (C-F) The osteoblastic genes expression, ARS and ALP staining of MSCs with or without *Usp18* or *Usp21* knockdown after 8 days of osteoblastic differentiation. (G and I) The osteoclastic genes expression of BMMs with or without *Usp21* or *Usp4* deletion after 3 days of osteoclastic differentiation. (H and J) The TRAP staining of multinucleated osteoclasts formation of BMMs with or without *Usp21* or *Usp4* deletion after 5 days of osteoclastic differentiation. *$P<0.05$, **$P<0.01$, ***$P<0.001$. P-values were analyzed by one-way ANOVA in (A) and two-tailed t tests in (B, C, E, G, I). All data are representative of two independent experiments.

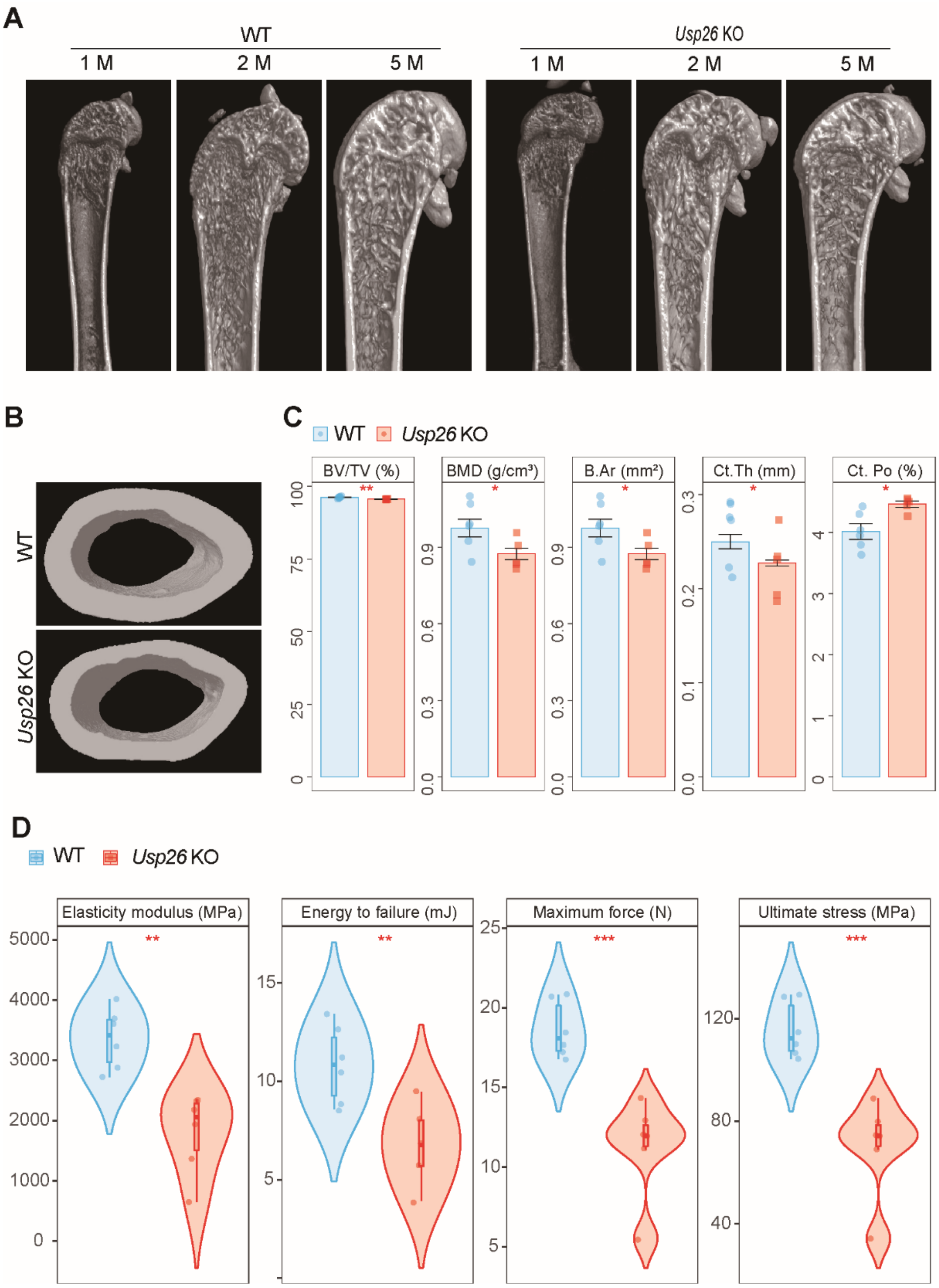

Fig. S2. ***Usp26*$^{-/-}$ mice have reduced bone mass and poor bone strength.** (A) Representative micro-CT images of femur bones of 1-, 2- and 5-month-old *Usp26*$^{-/-}$ mice and their WT littermates (*n*=5). (B) Representative micro-CT images of cortical bone from the femoral metaphysis of 5-month-old *Usp26*$^{-/-}$ mice and their WT littermates (*n*=6). (C) The cortical

bone volume (BV/TV, %), bone mineral density (BMD, g/cm^3), cotical thickness (Cb.Th, mm), total cross sectional cortical bone area (B. Ar, mm^2), and cortical porosity (Ct.Po, %) were determined by micro-CT analysis (*n*=6). (D) Maximum force and elasticity modulus analyzed using three-point bending test, including elasticity modulus (MPa), energy to failure (mJ), maximum force (N), and ultimate stress (MPa) (*n*=6). $*P<0.05$, $**P<0.01$, $***P<0.001$. *P*-values were analyzed by one-tailed *t* tests. All data are representative of two to three independent experiments.

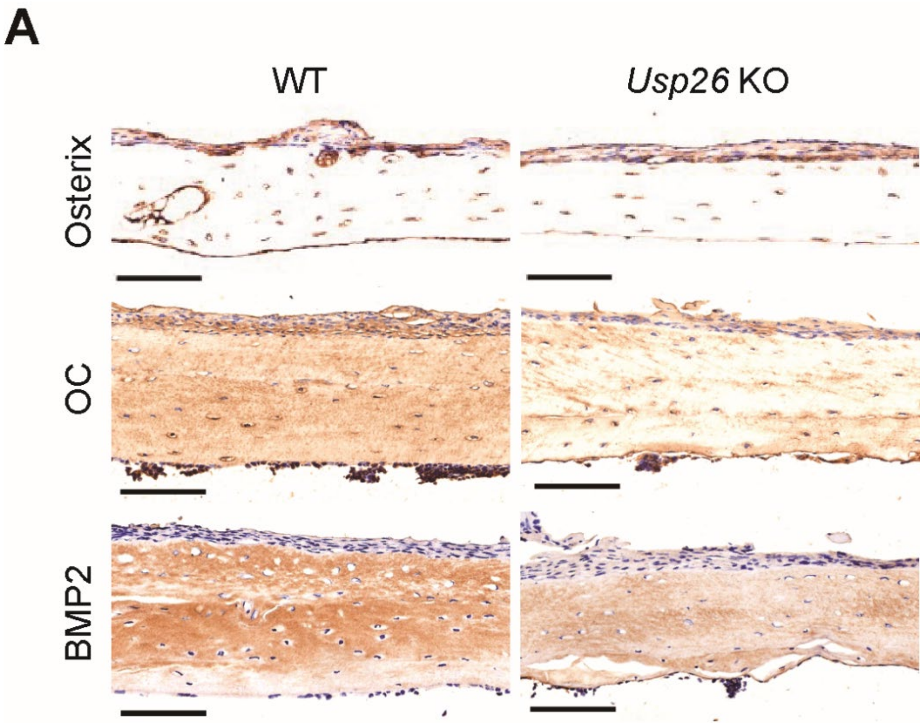

Fig. S3. (A) Osterix, OC and BMP2 immunohistochemical staining in femurs of 5-month-old *Usp26*$^{-/-}$ mice and their wild-type (WT) littermates (*n*=6). Scale bars represent 100 μm. All data are representative of two independent experiments.

1 matqadlmel dmamepdrka avshwqqqsy ldsgihsgat ttapslsgkg npeeedvdts
61 qvlyeweqgf sqsftqeqva didgqyamtr aqrvraamfp etldegmqip stqfdaahpt
121 nvqrlaepsq mlkhavvnli nyqddaelat raipeltkll ndedqvvvnk aavmvhqlsk
181 keasrhaimr spqmvsaivr tmqntndvet arctagtlhn lshhreglla ifksggipal
241 vkmlgspvds vlfyaittlh nlllhqegak mavrlagglq kmvallnktn vkflaittdc
301 lqilaygnqe skliilasgg pqalvnimrt ytyekllwtt srvlkvlsvc ssnkpaivea
361 ggmqalglhl tdpsqrlvqn clwtlrnlsd aatkqegmeg llgtlvqllg sddinvvtca
421 agilsnltcn nyknkmmvcq vggiealvrt vlragdredi tepaicalrh ltsrhqeaem
481 aqnavrlhyg lpvvvkllhp pshwplikat vglirnlalc panhaplreq gaiprlvqll
541 vrahqdtqrr tsmggtqqqf vegvrmeeiv egctgalhil ardvhnrivi rglntiplfv
601 qllyspieni qrvaagvlce laqdkeaaea ieaegatapl tellhsrneg vatyaaavlf
661 rmsedkpqdy kkrlsvelts slfrtepmaw netadlgldi gaqgealgyr qddpsyrsfh
721 sggygqdalg mdpmmehemg ghhpgadypv dglpdlghaq dlmdglppgd snqlawfdtd
781 l

Fig. S4. Sequences of the β-catenin protein and peptides identified by liquid chromatography-tandem mass spectrometry. The identified peptide sequences are shown in red.

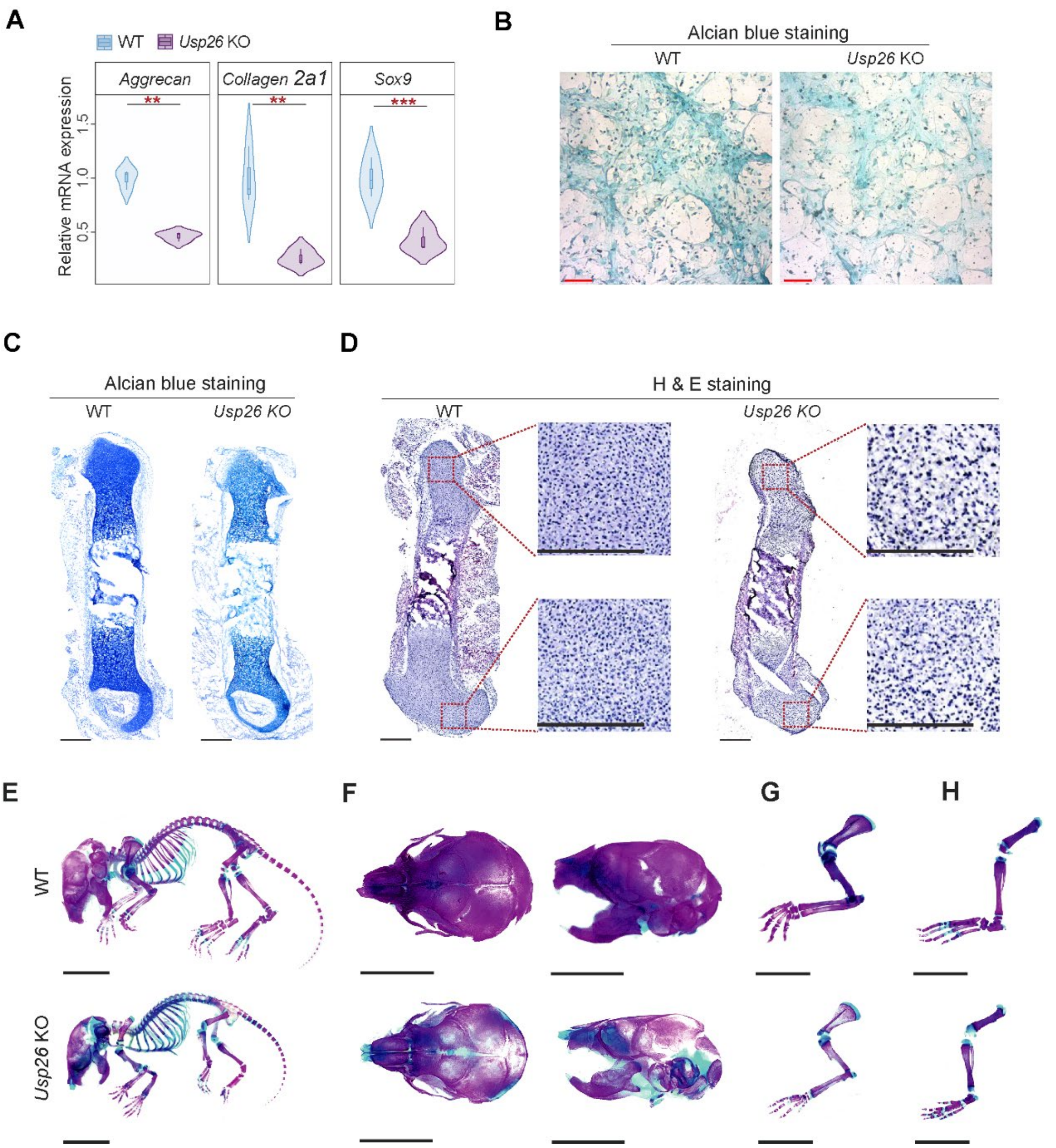

Fig. S5. *Usp26* deletion impaired chondrogenesis of MSCs, resulted in decreased chondrocyte formation and abnormal early skeletal development. (A) Quantification analysis of *Aggrecan*, *Collagen 2α1*, and *Sox9* expression in WT and *Usp26*$^{-/-}$ MSCs after 14 days of chondrogenic differentiation. (B) Alcian blue staining of WT and *Usp26*$^{-/-}$ MSCs after 14 days of chondrogenic differentiation. Scale bars represent 20 μm. (C and D) Alcian blue and H&E staining of femoral sections from E16.5 WT and *Usp26*$^{-/-}$ mice (*n*=5). Scale bars represent 20 μm. (E-H) Alcian blue/alizarin

red staining of the whole skeleton (E), calvaria (F), forelimb (G), and hindlimb (H) from 1-week-old WT and *Usp26*$^{-/-}$ male littermates (n=3). Scale bars represent 1 cm. $**P<0.01$, $***P<0.001$. P-values were analyzed by one-tailed t tests. All data are representative of two independent experiments.

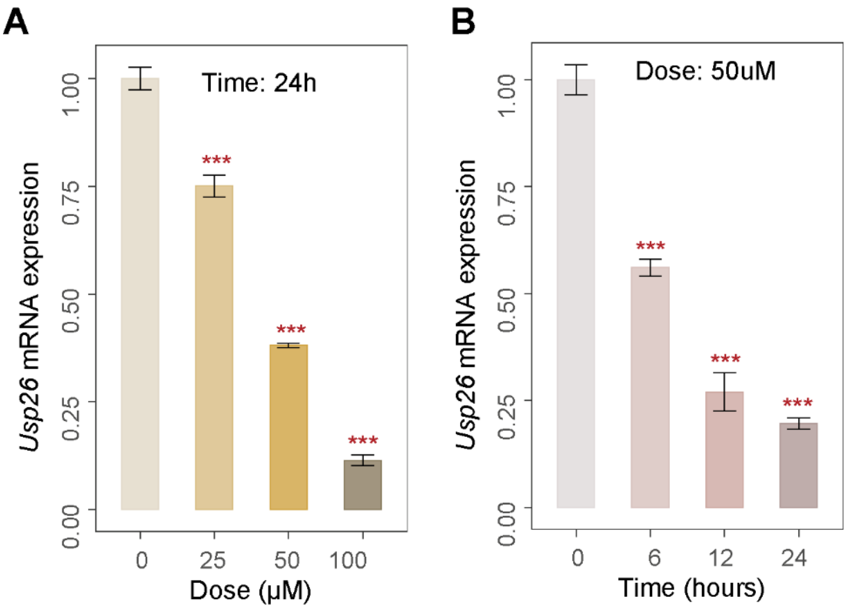

Fig. S6. Oxidative stress inhibited *Usp26* expression in MSCs. (A and B) Dose- and time-dependent expression of *Usp26* in MSCs exposed to H_2O_2. $***P<0.001$. P-values were analyzed by one-way ANOVA. All data are representative of two independent experiments.

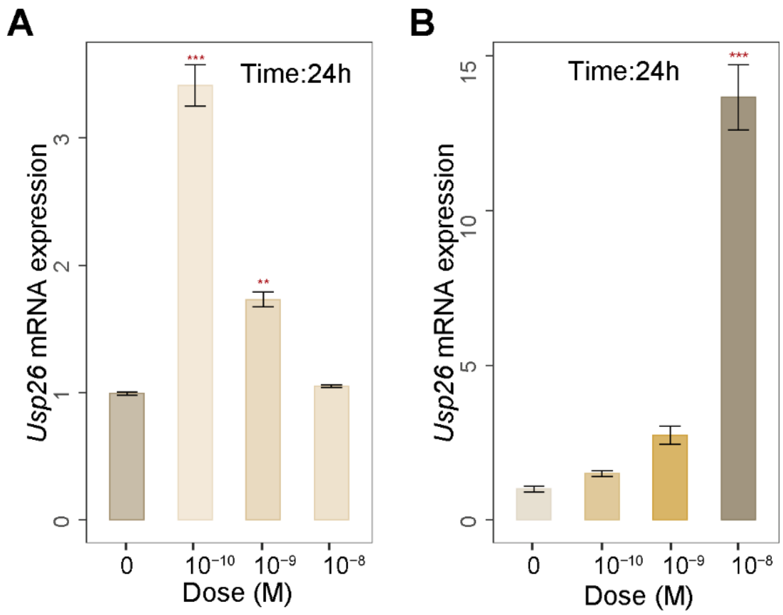

Fig. S7. Estrogen induced *Usp26* expression in BMMs and MSCs. (A and B) Dose- dependent expression of *Usp26* in BMMs (A) and MSCs (B) exposed to estrogen. $^{**}P<0.01$, $^{***}P<0.001$. *P*-values were analyzed by one-way ANOVA. All data are representative of two independent experiments.

Table S1. Primer sequences for real time-PCR

Gene		Primer sequence（5’- 3’）
Trap	FORWARD	CACTCCCACCCTGAGATTTGT
	REVERSE	CATCGTCTGCACGGTTCTG
C-Fos	FORWARD	CGGGTTTCAACGCCGACTA
	REVERSE	TTGGCACTAGAGACGGACAGA
Cathepsin k	FORWARD	GAAGAAGACTCACCAGAAGCAG
	REVERSE	TCCAGGTTATGGGCAGAGATT
DC-STAMP	FORWARD	GGGGACTTATGTGTTTCCACG
	REVERSE	ACAAAGCAACAGACTCCCAAAT
V-atpase α3	FORWARD	CACAGGGTCTGCTTACAACTG
	REVERSE	CGTCTACCACGAAGCGTCTC
Nfatc1	FORWARD	GACCCGGAGTTCGACTTCG
	REVERSE	TGACACTAGGGGACACATAACTG
Osterix	FORWARD	ATGGCGTCCTCTCTGCTTG
	REVERSE	TGAAAGGTCAGCGTATGGCTT
Runx2	FORWARD	CCACCACTCACTACCACACG
	REVERSE	GGACGCTGACGAAGTACCAT
Osteocalcin	FORWARD	CCTGACTGCATTCTGCCTCT
	REVERSE	AGGTAGCGCCGGAGTCTATT
Bmp-2	FORWARD	GAAGCCAGGTGTCTCCAAGA
	REVERSE	GGATGTCCTTTACCGTCGTG
Alp	FORWARD	GACAAGAAGCCCTTCACAGC
	REVERSE	ACTGGGCCTGGTAGTTGTTG
Col2α1	FORWARD	CCACACCAAATTCCTGTTCA
	REVERSE	ACTGGTAAGTGGGGCAAGAC
Aggrecan	FORWARD	AGGACCTGGTAGTGCGAGTG
	REVERSE	GCGTGTGGCGAAGAA
Sox9	FORWARD	CCACGGAACAGACTCACATCTCTC
	REVERSE	CTGCTCAGTTCACCGATGTCCACG
Usp1	FORWARD	ACAGATGAACTTGCTACACAGC
	REVERSE	AGGAGTTGGCATGTTTCTTGAA
Usp2	FORWARD	CACAGCAGTCTTTTCCCTTCG
	REVERSE	CATCTGTGTAGCGGGACGAT
Usp3	FORWARD	ACAGTGTGTATGGATTGCAGTAG
	REVERSE	CCTGTCCGCTGTAAAGGCT
Usp4	FORWARD	GGGGCGCAGTGGTATCTTATT
	REVERSE	GAAACAGGTTATGCTCCCCGA
Usp5	FORWARD	TGTCAGTGTTACCGACGATCC
	REVERSE	CCGGCGTGTCGAAAGAGAAA
Usp6	FORWARD	TCCGACCAGGATGTAGCACTC
	REVERSE	CTTCCCAGGGCTCGATCTCT
Usp7	FORWARD	CCACAAGGAAAACGACTGGG
	REVERSE	GTAACACGTTGCTCCCTGATT

Usp8	FORWARD	CTGCTAGTTGGATTGAAGCAAAC
	REVERSE	AGTGCATCTTTCAGACTCCGTA
Usp9	FORWARD	AGCCATCCGAATGGTTCGC
	REVERSE	ACAAGTGTTTTCCACGAAATGC
Usp10	FORWARD	GTCATCGAACCTAGTGAGGGG
	REVERSE	CCAAGGATAAATTCGGGTGCC
Usp11	FORWARD	GGCTGTATCAACAATGCTGGG
	REVERSE	TCATCTCCTTCTAGCAGTCTCTC
Usp12	FORWARD	CAGTCTCCAAATTCGCCTCCA
	REVERSE	GTGCTCGTTGACCGGAAACT
Usp13	FORWARD	CACATGGGAAAACGAAGTGCC
	REVERSE	GCCGTCAGTCAGATTCAACCA
Usp14	FORWARD	ATGCCACTCTACTCTGTTACAGT
	REVERSE	AACACCATTGGAGGTTCATCAG
Usp15	FORWARD	GACGCTGCTCAAAACCTCG
	REVERSE	CGATGGGTCCAGGATAGACATT
Usp16	FORWARD	CTGCCAAGACTGTAAGACTGAC
	REVERSE	GGTGTCGTGTAGTGCTTCAAG
Usp17	FORWARD	CCAGAAGAGACTGGAGGGGA
	REVERSE	ACCACCATGTCTCCAAAGACC
Usp18	FORWARD	CAGGAGTCCCTGATTTGCGT
	REVERSE	CAGAGGCTTTGCGTCCTTATC
Usp19	FORWARD	TGAACCAGAGCAGTGTACGTT
	REVERSE	CTGCACCTCGTGTAGCAGG
Usp20	FORWARD	TGGACTGCATAGGGGAGGTG
	REVERSE	ACTGGCAGGTTCCCTTAGATT
Usp21	FORWARD	AACTCCATGTTACGACCTTTGC
	REVERSE	AAGGGGACCTCTAGGACGAGA
Usp22	FORWARD	TCTTTCTGTCGGATAGGCACC
	REVERSE	GCCCTGAGTAAAACTCCTGGA
Usp24	FORWARD	GCTGGAAAGCCGCGTTTTG
	REVERSE	CAAGTCTGGCTAAGTAGGTGGA
Usp25	FORWARD	GGAGGAGACAGGCTATTACCA
	REVERSE	TCAAGGCAATCGCTCTCTGAA
Usp26	FORWARD	CTCAAGTCCAGATGTGGAGTGC
	REVERSE	CTGGTCTTCGCCATAGGTTTG
Usp27	FORWARD	CGACCAAACCTGAACTAGAACTT
	REVERSE	CGTAAGCCGATGGTAAAGCTG
Usp28	FORWARD	GGGTCCGAGAAGGAAAGCC
	REVERSE	CACGGAACGATCCGAAGGAAG
Usp29	FORWARD	TCCGCAGCACAAACAGGAG
	REVERSE	CTCACCACTAACCACTACGCC
Usp30	FORWARD	GGGAGTGATCGGTGGGATTG
	REVERSE	TCTTCCTCTCTGTAATGGGACC

Usp31	FORWARD	GGAGTACACCCCGCAACAC
	REVERSE	TGCTTTACAGCATGGTTGAGG
Usp32	FORWARD	AGTGAATCCGGGAGCTATGTT
	REVERSE	TTCCTGAGTGTATCTGGGACTTT
Usp33	FORWARD	GCGCTTGTCAGGACTGTAAAG
	REVERSE	CAGCCAACATAGGAGCACC
Usp34	FORWARD	GATATTGGTGGTCGTTCATGTGT
	REVERSE	TTGGCAAATTCGTAAAGGAAAGC
Usp35	FORWARD	GGGAAGAACATTGACAAGTGGA
	REVERSE	GCGAAACCTCGATCAAGATGC
Usp36	FORWARD	CCAACAGCGGCAATGCTATC
	REVERSE	CATCGCATCAATGGTGTACCG
Usp37	FORWARD	TGCAGACTGGGATTACAAAGTG
	REVERSE	ACGAGCAGGCTGACTCTATTG
Usp38	FORWARD	GCCCCTCAAGCGGATGATT
	REVERSE	GGGTCGTCAGGTCAAACATGG
Usp39	FORWARD	GTCACTGCCCGTACTTGGATA
	REVERSE	GTATGCGTTGATGTGCGAGAG
Usp40	FORWARD	TGACCGACTGGTTAAAGCAGC
	REVERSE	GCTAGTATCCTTGTAGCGTTCAC
Usp42	FORWARD	AGGCGGTCTCACCTGAAGA
	REVERSE	CACTGGCCCTAATGGAAGTGT
Usp43	FORWARD	GACTTGTCCACACTGCCTAAAA
	REVERSE	AGTGACACTCAAGAATCTCGTCT
Usp44	FORWARD	AATGGTACTGTATGGTCTGCAAC
	REVERSE	TTCAGTGCGTGCTCTTGGATG
Usp45	FORWARD	ATGCGGGTAAAAGATCCATCAAA
	REVERSE	ACGTTAGACCTACAGCAATGTCA
Usp46	FORWARD	ATGACTGTCCGAAACATCGCC
	REVERSE	TTGACCAATCCGAAGTAGTGTTC
Usp47	FORWARD	GATGTGATTCCCTTGGATTGCT
	REVERSE	AACCCCATTGGTGTATCTTCTTC
Usp48	FORWARD	TGTGGGCTTGACTAACCTGG
	REVERSE	AAGCTCCAAGTTGAGGAACCA
Usp49	FORWARD	AGTTCCGGGAATGTTTCCTGA
	REVERSE	CTCCTTACTGACAACTCTGCG
Usp50	FORWARD	GCCTACCTGATGACAGATATGTG
	REVERSE	TGCCAACAGCCGACAGAAAT
Usp51	FORWARD	CCCACGTCGGAGAACTTAACA
	REVERSE	GACAAGCAGCCGGGATAGAAG
Usp52	FORWARD	CAGAGGTGGAACCGCTTCAT
	REVERSE	TGAGCAGAAACTGCCTCCAG
Usp53	FORWARD	AAGCCTAGCGGCAATCTTGG
	REVERSE	GTTCTGCCCTGGCTCGTTTA

Usp54	FORWARD	CCTGAGCTCGAATGGGTTGT
	REVERSE	GAGACACCACAAGCCGAGAA
β-actin	FORWARD	GGCTGTATTCCCCTCCATCG
	REVERSE	CCAGTTGGTAACAATGCCATGT

Table S2. shRNA oligonucleotides

	5’	Stem	Loop	Stem	3’
***Usp18* shRNA**					
Usp18-shRNA-1 F	CCGG	GATGGCTGACTTTGGTCATTA	CTCGAG	TAATGACCAAAGTCAGCCATC	TTTTTG
Usp18-shRNA-1 R	AATTCAAAAA	GATGGCTGACTTTGGTCATTA	CTCGAG	TAATGACCAAAGTCAGCCATC	——
Usp18-shRNA-2 F	CCGG	GCTGCATTTCAACGAGAATAA	CTCGAG	TTATTCTCGTTGAAATGCAGC	TTTTTG
Usp18-shRNA-2 R	AATTCAAAAA	GCTGCATTTCAACGAGAATAA	CTCGAG	TTATTCTCGTTGAAATGCAGC	——
Usp18-shRNA-3 F	CCGG	CAGGTTCTGAAGCTGACTCAT	CTCGAG	ATGAGTCAGCTTCAGAACCTG	TTTTTG
Usp18-shRNA-3 R	AATTCAAAAA	CAGGTTCTGAAGCTGACTCAT	CTCGAG	ATGAGTCAGCTTCAGAACCTG	——
***Usp21* shRNA**					
Usp21-shRNA-1 F	CCGG	ATGGCTCCTTCCACATGATAT	CTCGAG	ATATCATGTGGAAGGAGCCAT	TTTTTG
Usp21-shRNA-1 R	AATTCAAAAA	ATGGCTCCTTCCACATGATAT	CTCGAG	ATATCATGTGGAAGGAGCCAT	——
Usp21-shRNA-2 F	CCGG	GTGCTCCATCTGAACCGATTT	CTCGAG	AAATCGGTTCAGATGGAGCAC	TTTTTG
Usp21-shRNA-2 R	AATTCAAAAA	GTGCTCCATCTGAACCGATTT	CTCGAG	AAATCGGTTCAGATGGAGCAC	——
Usp21-shRNA-3 F	CCGG	CCTAAGGTTAAGTCGCCCTCG	CTCGAG	CGAGGGCGACTTAACCTTAGG	TTTTTG
Usp21-shRNA-3 R	AATTCAAAAA	CCTAAGGTTAAGTCGCCCTCG	CTCGAG	CGAGGGCGACTTAACCTTAGG	——
**Negative control**					
shRNA Scramble F	CCGG	CCTAAGGTTAAGTCGCCCTCG	CTCGAG	CGAGGGCGACTTAACCTTAGG	TTTTTG
shRNA Scramble R	AATTCAAAAA	CCTAAGGTTAAGTCGCCCTCG	CTCGAG	CGAGGGCGACTTAACCTTAGG	——

Table S3. siRNA oligonucleotides

	sense (5‘-3’)	antisense (5’-3’)
Usp4 siRNA-1	CGCAGUGGUAUCUUAUUGATT	UCAAUAAGAUACCACUGCGTT
Usp4 siRNA-2	GAGCAAGCUAGACAACACUTT	AGUGUUGUCUAGCUUGCUCTT
Usp4 siRNA-3	GCGUAAAGAAGAAGCCUUATT	UAAGGCUUCUUCUUUACGCTT
Negative control	UUCUCCGAACGUGUCACGUTT	ACGUGACACGUUCGGAGAATT
