# Supplementary material for: The osteoprotective role of USP26 in coordinating bone formation and resorption
Source: Cell Death Differ. 2022 Jan 29;29(6):1123–36. doi: 10.1038/s41418-021-00904-x (PMC9177963; doi:10.1038/s41418-021-00904-x)
Supplement: Supplementary file 3 — Detailed Author Contribution form [file 41418_2021_904_MOESM3_ESM.pdf]

DECLARATION OF CONTRIBUTIONS TO ARTICLE

ADMC

Manuscript Number: CDD-21-0559R

Journal Name: *Cell Death & Differentiation* (the 'Journal')

Proposed Title of the Contribution:

The osteoprotective role of USP26 in coordinating bone formation and resorption (the 'Contribution)

Author(s):

Changwei Li, Minglong Qiu, Leilei Chang, Jin Qi, Lianfang Zhang, Bernhard Ryffel, Lianfu Deng (the 'Authors')

For all *CDD* articles, each person named as an author in the published version must be able to show he or she has contributed substantially to the article.

Authorship credit should be based on 1) substantial contributions to conception and design, acquisition of data, or analysis and interpretation of data; 2) drafting the article or revising it critically for important intellectual content; and 3) final approval of the version to be published. Authors should meet conditions 1, 2 and 3.

Any person who cannot be shown to have made a substantial contribution to the article cannot be listed as an author in the final version. The name of any person who is deemed to have made a minor contribution can, however, appear in the Acknowledgments section of the article.

Please complete the table below to indicate the contributions of all named authors to the manuscript.

Author Full Name:	Specification of Contribution to the Manuscript:
Changwei Li	Conceived and designed the experiments; Performed the experiment; Analyzed the data; Contributed reagents/materials/analysis tools; Wrote, review and revision of the manuscript
Minglong Qiu	Performed the experiments
Leilei Chang	Performed the experiments; Analyzed the data
Jin Qi	Performed the experiments
Lianfang Zhang	Contributed reagents/materials/analysis tools
Bernhard Ryffel	Conceived and designed the experiments; Contributed reagents/materials/analysis tools; Wrote, review and revision of the manuscript
Lianfu Deng	Conceived and designed the experiments; Contributed reagents/materials/analysis tools

Please complete the table below to indicate the contributions of all named authors to the figures.

Figure 1:

C.L. generated the data and prepared Fig 1A, B, C, D, G, H, I; L.C. generated the data and prepared Fig 1E, F, J, K; C.L. assembled the figure.

Figure 2:

J.Q. generated the data and prepared Fig 1A, G, H, K, L; C.L. generated the data and prepared Fig 1B, C, E, F, J; L.C. generated the data and prepared Fig 1D, I; C.L. assembled the figure.

Figure 3:

C.L. generated the data and prepared Fig 1A, B, C, E, F, I, J, K, L, M, N; L.C. generated the data and prepared Fig 1G, H; J.Q. generated the data and prepared Fig 1D; C.L. assembled the figure.

Figure 4:

C.L. generated the data and prepared Fig 1A, B, C, D, E, J, K, M, N, R, S; L.C. generated the data and prepared Fig 1G, H, L, O, P, Q; J.Q. generated the data and prepared Fig 1F, I; C.L. assembled the figure.

Figure 5:

M.Q. drafted Fig 1A, H; C.L. generated the data and prepared Fig 1B, C, D, E, F, I, J, K; L.C. generated the data and prepared Fig 1G; C.L. assembled the figure.

Figure 6:

M.Q. generated the data and prepared Fig 1A, B, C; J.Q. generated the data and prepared Fig 1D, M, N, O; L.C. generated the data and prepared Fig 1E, F; C.L. generated the data and prepared Fig 1G, H, I, J, K, L; C.L. assembled the figure.

Signed for and on behalf of the Author(s):	Print Name:	Date:
Changwei Li	Changwei Li	November 4, 2021
